# Synthesis and biochemical evaluation of 17-*N*-beta-aminoalkyl-4,5α-epoxynormorphinans

**DOI:** 10.1038/s41598-023-46317-3

**Published:** 2023-11-20

**Authors:** Ferenc Ötvös, Edina Szűcs, Ákos Urai, István Köteles, Pál T. Szabó, Zsuzsanna Katalin Varga, Dávid Gombos, Sándor Hosztafi, Sándor Benyhe

**Affiliations:** 1 Institute of Biochemistry, HUN-REN Biological Research Centre, Temesvári Krt. 62, 6726 Szeged, Hungary; 2 Institute of Pharmaceutical Chemistry, Semmelweis Medical University, Hőgyes Endre Utca 9, 1092 Budapest, Hungary; 3 Department of Chemistry and Molecular Biology, University of Gothenburg, Medicinaregatan 19, 41390 Göteborg, Sweden; 4 Research Centre for Natural Sciences, MS Metabolomics Research Laboratory, Magyar Tudósok Krt. 2, 1117 Budapest, Hungary; 5 Theoretical Medical Doctoral School, Faculty of Medicine, University of Szeged, 6726 Szeged, Hungary

**Keywords:** Drug discovery and development, Computational models

## Abstract

Opiate alkaloids and their synthetic derivatives are still widely used in pain management, drug addiction, and abuse. To avoid serious side effects, compounds with properly designed pharmacological profiles at the opioid receptor subtypes are long needed. Here a series of 17-*N*-substituted derivatives of normorphine and noroxymorphone analogues with five- and six-membered ring substituents have been synthesized for structure–activity study. Some compounds showed nanomolar affinity to MOR, DOR and KOR in in vitro competition binding experiments with selective agonists [^3^H]DAMGO, [^3^H]Ile^5,6^-deltorphin II and [^3^H]HS665, respectively. Pharmacological characterization of the compounds in G-protein signaling was determined by [^35^S]GTPγS binding assays. The normorphine analogues showed higher affinity to KOR compared to MOR and DOR, while most of the noroxymorphone derivatives did not bind to KOR. The presence of 14-OH substituent resulted in a shift in the pharmacological profiles in the agonist > partial agonist > antagonist direction compared to the parent compounds. A molecular docking-based in silico method was also applied to estimate the pharmacological profile of the compounds. Docking energies and the patterns of the interacting receptor atoms, obtained with experimentally determined active and inactive states of MOR, were used to explain the observed pharmacological features of the compounds.

## Introduction

Pain transmission is partially regulated by the neuronal membrane-located opioid receptors which are important members of Class A G-protein coupled receptors (GPCRs) possessing seven helical transmembrane domains^1–5^. Opioid receptors are expressed in both the central and peripheral nervous systems. Activation of the three classical types of opioid receptors (mu-, delta- and kappa-opioid receptors, referred as MOR, DOR and KOR, respectively) leads to inhibition of the adenylyl cyclase resulting in hyperpolarization in the cell and inhibits neurotransmitter release^6,7^.

Opioid receptors have their own endogenous peptide ligands^8^. Besides those numerous exogenous opiates were discovered^9^. Among them biphalin and derivatives are particularly interesting because they have many different physiological effects^10,11^. Fentanyl and its derivatives^12^ are among the most important synthetic opiates used in analgesia. Morphine and its semi synthetic derivatives traditionally used in pain medication. Due to their importance this study is devoted to investigate morphine derivatives.

Morphine is typically a MOR selective compound^13^. The analgesic effect is altered when injected directly to the spinal cord, whereby the release of neurotransmitters is inhibited from nociceptive afferents or by hyperpolarization of cells in the substantia gelatinosa where the afferents terminate. Morphine was first isolated by the German pharmacist Friedrich Sertürner from poppy in 1805^14^. Further research has confirmed that poppy contains nearly 50 alkaloids from which morphine is still the most used drug in pain medication. It acts directly on the central nervous system resulting in pain relief and analgesia. Besides, it has serious side effects including decreased respiratory rate, low blood pressure and also has a high potential for addiction and abuse^15–18^. Due to the side effects, there has been an ongoing effort to find new compounds with improved affinity and selectivity profile to obtain more effective drugs with decreased side effects.

The substitution on the nitrogen of morphine derivatives is critical to the degree and type of activity displayed by the N-substituted-norcompound. The size of the N-substituent can confer the compound’s potency and its agonist or antagonist properties. Increasing the size of the N-substituent to three to five carbons yields derivatives which are antagonists at some or all opioid receptor types. Allyl, cyclopropylmethyl and normal propyl are the characteristic antagonist substituents, but in most cases these analogues are mixed agonist-antagonists. Replacement of the N-methyl group of oxymorphone results in pure antagonists: for example, N-allyl-noroxymorphone (Naloxone) or N-cyclopropylmethyl-noroxymorphone (Naltrexone)^19,20^). The pure antagonists may have variable affinity for the opioid receptors but they have no intrinsic analgesic activity. In summary the nature of the N-substituent is crucial for the modulation of affinity and selectivity for the mu, kappa and delta opioid receptors as well as agonist and antagonist functional activity.

**Figure 1:**
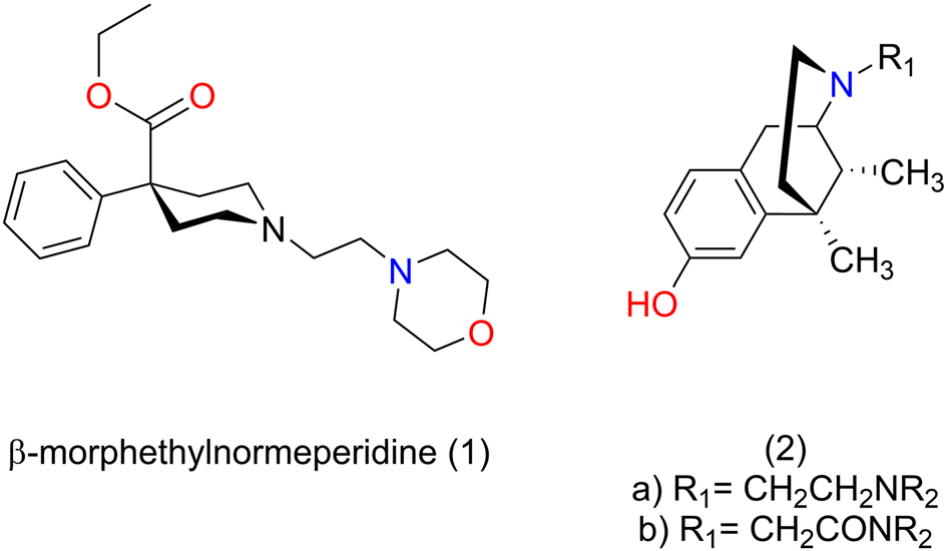
Truncated morphinan derivative scaffolds. Legend: (1) Fentanyl-like, (2) pentazocine-like.

Alterations in the morphinan scaffold were also extensively investigated leading to new class of opiates (Fig. 1). It was reported that replacement of N-methyl group of meperidine with β-morpholinoethyl group (1) resulted in an increase of analgesic activity^21,22^.

Ronsisvalle et al. prepared β-aminoethyl-N-substituted derivatives of (−)-normetazocine (2) by alkylation with β-chloroethyl-piperidines (and pyrrolidines and morpholines)^23–25^. (−)-Normetazocine was also alkylated with chloroacetamides which were prepared in the reaction of chloroacetyl chloride with cyclic secondary amines. Opioid receptor binding of these new derivatives was evaluated and some compounds displayed different μ, κ, and δ receptor selectivity profiles depending on the N-substituent.

Previously our research team has studied the structure–activity relationships of N-substsituted-4,5-epoxynormorphinans^26–28^ and continuing these projects we designed the syntheses of N-β-aminoalyl-4,5-epoxynormorphinans and N-acetamido-4,5-epoxynormorphinans. Because the N-β-aminoalkyl-4,5-epoxynormorphinans contain two basic nitrogens our aim was to study the influence of the β-nitrogen on receptor binding and on analgesic activity. The N-acetamido-substituents can also affect the receptor binding.

We designed the N-alkylation of 4,5-epoxynormorphinans by chloroethylamines and chloroacetamides to assess the agonist/antagonist profiles of these derivatives and also the binding selectivity at the opioid receptors. The second nitrogen atom of the N-alkyl group is a part of a heterocyclic ring system, such as morpholine, piperidine and pyrrolidine. In this project we can study potencies of two sets of compounds, the first group of compounds possess another basic nitrogen and in the second group the basicity of the N-substituent nitrogen is blocked because of the amide group formation.

The aim of the present study was to investigate the binding and pharmacological properties of the **4a** and **7a** derivatives, i.e. comparing the normorphine/noroxymorphone scaffolds too, for the opioid receptors (MOR, DOR and KOR) in rat and guinea pig brain membrane preparations. The binding affinities and receptor selectivity for MOR, DOR and KOR were determined by radioligand displacement assays. The pharmacological profile (agonist, partial agonist, antagonist or inverse agonist in the G-protein activation) of the ligands through all three opioid receptors was determined by [^35^S]GTPγS ([^35^S]GTPgammaS) functional binding assay.

Due to the fact that most of the unwanted side-effects and the abuse potential of opiates are related to MOR, an in silico pharmacological profiling of the newly synthesized compounds was also attempted on MOR. Although numerous efforts have been made to explain or predict the pharmacological profile of GPCR ligands^29^ using in silico methods, most of them rely on supervised models. Present study attempts to introduce unsupervised classification methods, principal component analysis and multiple component analysis, which both belong to exploratory data analysis methods.

## Results

### In vitro assays

#### Competition binding

**Figure 2:**
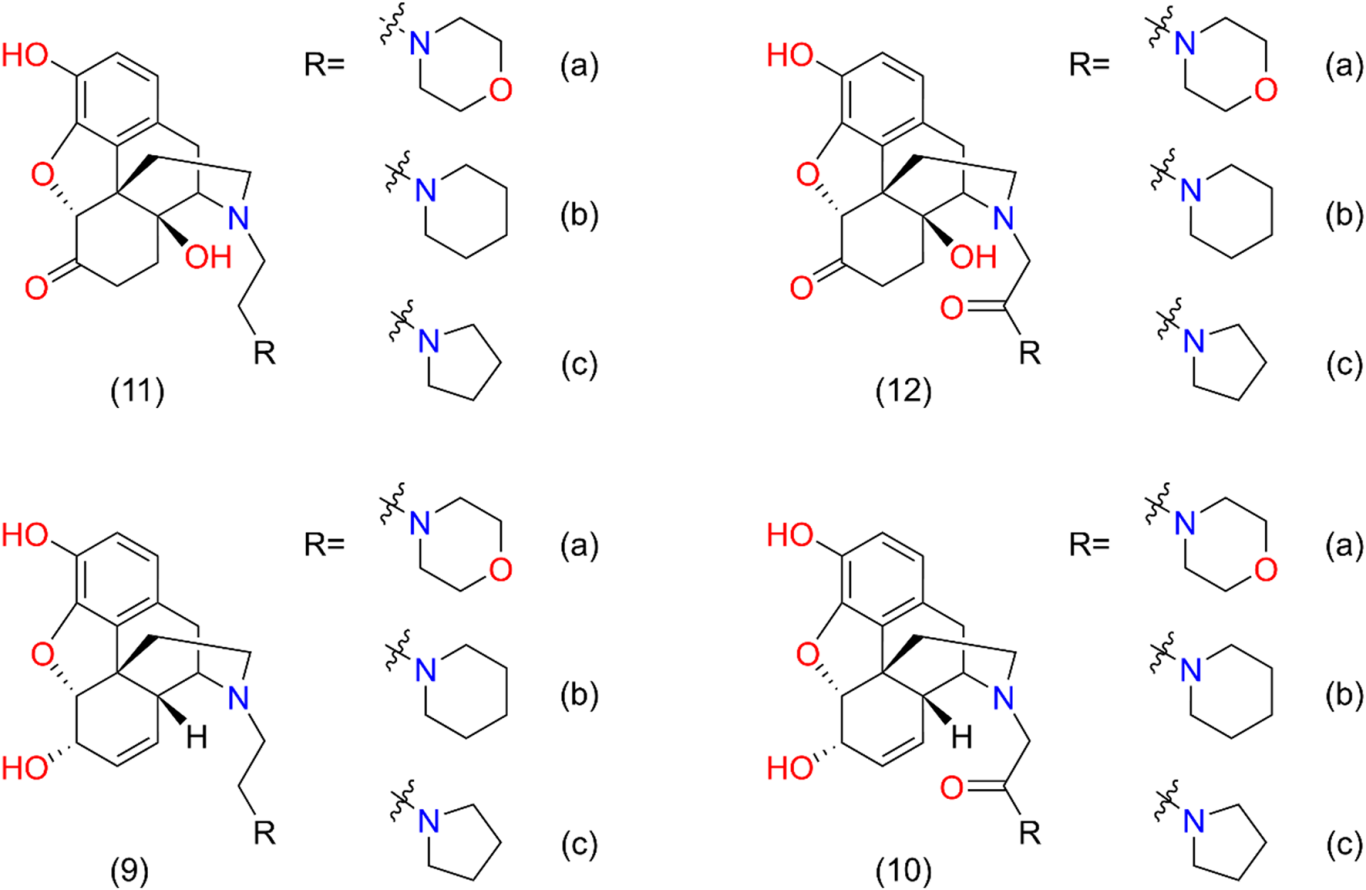
Derivatives used for the assays.

Analogues were characterized in [^3^H]DAMGO and [^3^H]Ile^5,6^-deltorphin II displacement experiments in rat brain homogenates and in [^3^H]HS665 displacement in guinea pig brain homogenates. Reference data of the radioligands were determined in homolog displacement measurements. Ligands showed lower equilibrium binding affinity (K_i_ value) in μ-opioid system and in δ-opioid system than DAMGO and Ile^5,6^-deltorphin II, respectively (Table 1, and Supplementary Fig. S1A–D). In κ-opioid system the **7a** noroxymorphone derivatives (**11a, b, c** and **12a, b, c**) did not show binding affinity except **11a** and **11b** (Supplementary Fig. S1E). The **4a** normorphine derivatives (**9a, b, c** and **10a, b, c**) showed similar K_i_ values in competition with the selective KOR agonist HS665 except **9b** and **10b** (Supplementary Fig. S1F) (chemical structures are shown in Fig. 2 and in the “Materials and methods” section).

**Table 1:** Displacement of [^3^H]DAMGO, [^3^H]Ile^5,6^-deltorphin II and [^3^H]HS665 by DAMGO, Ile^5,6^-deltorphin II, HS665 and the morphine analogues in membranes of rat and guinea pig brain.

Ligand	MOR^a^	DOR^a^	KOR^b^	Selectivity for μ site	
	Ki ± S.E.M. (nM)			K_iδ_/K_iμ_	K_iκ_/K_iμ_
DAMGO	2.7 ± 1.1	n.d.^c^	n.d.^c^	n.d.^c^	n.d.^c^
Ile^5,6^-deltorphin II	n.d.^c^	9.5 ± 0.9	n.d.^c^	n.d.^c^	n.d.^c^
HS665	n.d.^c^	n.d.^c^	2.1 ± 0.7	n.d.^c^	n.d.^c^
Naloxone	5.3 + 2.2	14.4 + 2.4	5.4 + 1.5	2.72	1.02
11a	72.0 ± 3.1	121.4 ± 12.2	2.7 ± 1.1	1.69	0.04
11b	75.2 ± 3.1	205.7 ± 44.9	39.6 ± 4.9	2.73	0.52
11c	74.5 ± 3.3	168.7 ± 11.8	N.B.	2.26	n.d.^c^
12a	684.0 ± 32.7	125.3 ± 39.6	N.B.	0.18	n.d.^c^
12b	1495.0 ± 612.0	1141.1 ± 532.0	N.B	0.76	n.d.^c^
12c	133.3 ± 55.3	117.0 ± 36.5	N.B.	0.88	n.d.^c^
Morphine	4.1 ± 1.7	9417.4 ± 274.0	197.0 ± 15.9	2297	48.0
4a	5.1 ± 2.4	4066.6 ± 165.0	2.5 ± 1.2	797.4	0.49
9a	389.1 ± 17.0	25.3 ± 1.5	20.3 ± 4.2	0.07	0.05
9b	435.9 ± 18.8	44.9 ± 7.2	137.5 ± 42.3	0.10	0.32
9c	62.3 ± 2.6	34.1 ± 7.1	6.9 ± 4.6	0.55	0.11
10a	393.9 ± 186.0	N.B	3.6 ± 1.9	n.d.^c^	0.01
10b	1498.8 ± 664.0	> 10,000	N.B.	n.d.^c^	n.d.^c^
10c	521.4 ± 218.0	839.2 ± 99.9	10.7 ± 7.7	1.61	0.02

^a^Rat brain membrane, ^b^guinea pig brain membrane, ^c^not determined, N.B. no binding.

The IC_50_ values for the MOR, DOR and KOR according to the competition binding curves (see Supplementary Fig. S1) were converted into equilibrium inhibitory constant (K_i_) values, using the Cheng–Prusoff equation^30^.

#### G-protein activation by functional GTPγS binding assay

The effect of the ligands on G-protein activation was investigated in functional [^35^S]GTPγS binding assays in rat brain membranes (Supplementary Fig. S2). **7a** derivatives did not produce a dose-dependent increase (Supplementary Fig. S2A). The **4a** derivatives stimulated G-protein activity, **9c** and **10a** had similar efficacy (E_max_) compared to morphine and **4a**, the other compounds showed lower efficacy (Table 2).

**Table 2:** The maximal G-protein efficacy (E_max_) of naloxone, morphine, **4a** and the derivatives of **4a** and **7a** in [^35^S]GTPγS binding assays in rat brain membrane homogenates.

Ligand	Maximal stimulation (efficacy)	Potency
	E_max_ ± S.E.M. (%)	Log EC_50_ ± S.E.M.
Naloxone	101.3 ± 0.4	n.d.^a^
11a	98.5 ± 1.2	n.d.^a^
11b	106.9 ± 1.9	n.d.^a^
11c	106.8 ± 2.7	n.d.^a^
12a	99.3 ± 1.2	n.d.^a^
12b	105.1 ± 1.9	n.d.^a^
12c	100.8 ± 3.8	n.d.^a^
Morphine	134.2 ± 1.5	− 7.21 ± 0.12
4a	145.8 ± 5.0	− 6.21 ± 0.24
9a	124.5 ± 2.1	− 7.87 ± 0.28
9b	132.0 ± 4.6	− 5.86 ± 0.25
9c	153.2 ± 4.6	− 6.19 ± 0.18
10a	139.0 ± 2.4	− 7.00 ± 0.15
10b	120.8 ± 2.5	− 5.89 ± 0.25
10c	120.0 ± 2.2	− 8.94 ± 0.36

^a^Not determined.

The values were calculated according to the dose–response binding curves shown in Supplementary Fig. S2.

#### Inhibition

The receptor specific G-protein activation of **7a** analogues was investigated in the absence or presence of the selective agonists DAMGO, Ile^5,6^-deltorphin II and U-69,593 ((+)-(5α,7α,8β)-N-Methyl-N-[7-(1-pyrrolidinyl)-1-oxaspiro[4.5]dec-8-yl]-benzeneacetamide) for MOR, DOR and KOR, respectively (Supplementary Fig. S3A and B). The derivatives reversed the agonist effect of DAMGO and Ile^5,6^-deltorphin II to the basal activity, **12a** significantly decreased the efficacy of DAMGO, **12b** did not change significantly the effect of DAMGO and that of Ile^5,6^-deltorphin II (Table 3). In guinea pig brain membrane homogenates, the analogues did not significantly inhibit the effect of U-69,593, except **11a** and **11b** (Table 3).

**Table 3:** The maximal G-protein efficacy (E_max_) of **7a** analogues in the absence or presence of the selective agonists.

	Ligand^a^	MOR^a^	DOR^a^	Ligand^b^	KOR^b^
		Ligand + DAMGO	Ligand + Ile^5,6^-deltorphin II		Ligand + U-69,593
E_max_ ± S.E.M. (%)					
DAMGO	159.7 ± 0.9	n.d.^c^	n.d.^c^	n.d.^c^	n.d.^c^
Ile^5,6^-deltorphin II	153.5 ± 0.4	n.d.^c^	n.d.^c^	n.d.^c^	n.d.^c^
U-69,593	n.d.^c^	n.d.^c^	n.d.^c^	150.6 ± 2.8	n.d.^c^
Naloxone	101.3 ± 1.1	102.8 ± 1.3****	101.1 ± 2.1***	100.7 ± 1.6	101.1 ± 1.0***
11a	103.9 ± 4.1	99.9 ± 5.0**	99.3 ± 1.8**	99.4 ± 1.5	101.6 ± 1.8***
11b	103.9 ± 5.2	97.7 ± 3.9**	103.7 ± 3.6**	100.8 ± 0.8	101.2 ± 2.0***
11c	103.1 ± 3.8	104.2 ± 0.5***	106.8 ± 1.7**	101.5 ± 2.5	135.1 ± 4.1^NS^
12a	100.1 ± 1.8	119.8 ± 8.9*	96.9 ± 5.9**	99.4 ± 1.5	138.4 ± 3.4^NS^
12b	101.9 ± 1.1	154.3 ± 5.2^NS^	140.5 ± 5.1^NS^	101.4 ± 2.5	138.4 ± 0.8^NS^
12c	100.4 ± 4.0	100.8 ± 5.0**	103.5 ± 3.5**	100.3 ± 1.6	133.3 ± 4.5^NS^

^a^Rat brain membrane, ^b^guinea pig brain membrane, ^c^not determined.

Experimental data were processed by GraphPad Prism 5.0 using bar graphs. ^NS^not significant; *P < 0.05; **P < 0.01; ***P < 0.001; ****P < 0.0001 based on unpaired t-tests.

MOR agonist DAMGO and the selective DOR agonist Ile^5,6^-deltorphin II in rat brain membrane homogenates (Supplementary Fig. S3A) and in the absence or presence of the selective KOR agonist U-69,593 in guinea pig brain membrane homogenates (Supplementary Fig. S3B) in [^35^S]GTPγS binding assays. The values were calculated according to bar graphs in Supplementary Fig. S3.

Similarly, the effect of **4a** derivatives was investigated in the absence or presence of the selective antagonist cyprodime, NTI (naltrindole) and nor-BNI (norbinaltorphimine) for MOR, DOR and KOR, respectively (Supplementary Fig. S3C and D). Cyprodime reversed the agonistic effect of **10c**, **10a** and **9c** to the basal activity, while the efficacies of **9a**, **9b** and **10b** were not changed by cyprodime. NTI decreased the efficacy of **9a**, **9b** and **9c** to the basal activity, while the efficacies of **10a**, **10b** and **10c** were not changed by NTI in rat brain membrane homogenates. In guinea pig brain membrane homogenates nor-BNI significantly inhibited the effect of the ligands, except **9b**. **10b** did not activate G-protein at all (Table 4).

**Table 4:** The maximal G-protein efficacy (E_max_) of **4a** derivatives in the absence or presence of the selective antagonists.

	Ligand^a^	MOR^a^	DOR^a^	Ligand^b^	KOR^b^
		Ligand + cyprodime	Ligand + NTI		Ligand + Nor-BNI
E_max_ ± S.E.M. (%)					
Morphine	135.5 ± 2.4	100.1 ± 1.1***	133.0 ± 2.8^NS^	114.3 ± 3.4	100.6 ± 1.0*
4a	140.2 ± 0.7	96.3 ± 4.8**	127.9 ± 3.3^NS^	138.9 ± 0.3	100.7 ± 3.0**
9a	129.6 ± 3.1	114.9 ± 6.7^NS^	104.2 ± 3.7*	135.0 ± 1.9	103.4 ± 0.5**
9b	131.2 ± 1.3	118.6 ± 4.0^NS^	103.2 ± 2.1**	122.1 ± 2.6	109.3 ± 6.3^NS^
9c	149.3 ± 2.2	96.7 ± 5.9 **	106.6 ± 5.5 **	151.0 ± 1.9	95.4 ± 1.4 ***
10a	141.5 ± 2.7	107.2 ± 3.8**	138.2 ± 6.3^NS^	143.9 ± 1.6	99.9 ± 2.3***
10b	118.1 ± 1.5	117.1 ± 3.0^NS^	119.3 ± 3.9^NS^	103.1 ± 3.6	103.1 ± 1.9^NS^
10c	124.9 ± 4.0	99.4 ± 1.6**	111.2 ± 3.1^NS^	130.2 ± 1.0	102.5 ± 3.6**

^a^Rat brain membrane, ^b^guinea pig brain membrane.

Experimental data were processed by GraphPad Prism 5.0 using bar graphs. ^NS^: not significant; *P < 0.05; **P < 0.01; ***P < 0.001 based on unpaired t-tests.

#### Partial agonistic effects

The agonistic character of **4a** derivatives, which efficacy were significantly inhibited by the selective antagonists, were investigated whether they are agonists or partial agonists. In our system morphine was observed as partial agonist while **4a** proved to be a full agonist at MOR. Partial agonists were able to decrease the efficacy of the full agonists to their own stimulation level as we reported before^31^. Increasing concentrations of the partial agonists were investigated in the presence of 10 µM selective agonists producing maximal stimulation (Supplementary Fig. S4). The compounds investigated were able to inhibit the activation mediated by selective agonists, although with relatively low efficacy and potency (Table 5). This antagonizing effect, in the presence of the full agonists validates that **10a** and **10c** are indeed partial agonist ligands for MOR (Supplementary Fig. S4A), **9a** and **9b** for DOR (Supplementary Fig. S4B), and **9a**, **10a** and **10c** for KOR (Supplementary Fig. S4C, Table 5). **10b** was not tested due to its inactivity in the [^35^S]GTPγS binding assay, and **9c** was also not tested because it acted as full agonist on each opioid receptors.

**Table 5:** Partial agonists effect of **4a** analogues in the presence of selective agonists.

	Ligand^a^	MOR^a^	DOR^a^	Ligand^b^	KOR^b^
		Ligand (10^–10^–10^–5^ M) + 10 µM of DAMGO	Ligand (10^–10^–10^–5^ M) + 10 µM of + Ile^5,6^-deltorphin II		Ligand (10^–10^–10^–5^ M) + 10 µM of + U-69,593
E_max_ ± S.E.M. (%)					
DAMGO	159.5 ± 1.6	n.d.^c^	n.d.^c^	n.d.^c^	n.d.^c^
Ile^5,6^-deltorphin II	152.9 ± 1.5	n.d.^c^	n.d.^c^	n.d.^c^	n.d.^c^
U-69,593	n.d.^c^	n.d.^c^	n.d.^c^	149.9 ± 1.0	n.d.^c^
Morphine	135.5 ± 2.4^d^	135.4 ± 4.3	155.3 ± 1.7	114.3 ± 3.4 ^d^	131.0 ± 2.6
4a	140.2 ± 0.7^d^	158.9 ± 11.0	152.9 ± 0.7	138.9 ± 0.3^d^	154.2 ± 1.6
9a	129.6 ± 3.1^d^	n.d.^c^	138.0 ± 1.9	135.0 ± 1.9^d^	136.7 ± 2.5
9b	131.2 ± 1.3^d^	n.d.^c^	134.6 ± 3.1	122.1 ± 2.6^d^	n.d.^c^
10a	141.5 ± 2.7^d^	135.5 ± 8.2	n.d.^c^	143.9 ± 1.6^d^	139.4 ± 2.5
10c	124.9 ± 4.0^d^	139.5 ± 4.5	n.d.^c^	130.2 ± 1.0^d^	122.8 ± 8.0

^a^Rat brain membrane, ^b^guinea pig brain membrane, ^c^not determined, ^d^values taken from Table 4.

The values were calculated according to the dose–response binding curves shown in Fig. S4.

#### In silico studies

The interaction of the ligands with MOR was modelled by molecular docking together with numerous opiate compounds characterized experimentally. Because the known consensus binding mode involves a charged interaction between ASP147 and the ligand, the success of the docking calculations was characterized by the distance between the 17-N atom of the ligands and the ASP147 oxygen. The docked positions of the new compounds are shown in Supplementary Figs. S5 and S6.

The N17-ASP147 distances were determined in both the active and inactive state of the receptor for all 106 ligands involved in the modelling studies. Comparing the results with the 2.7–3.3 Å range accepted for hydrogen bonds, there are 45 (ca. 20%) outlying docked positions as shown in Supplementary Fig. S7. However, examining the ligand activity and receptor state reveals that 84.4% of the outliers, 38 out of 45, the ligand activity and the receptor state did not match. Mostly antagonists were not docked properly in the active receptor model and numerous issues were found for partial agonists in both receptor models: 46.6% of the not fitting ligands were partial agonists. Interestingly, the agonists were properly docked into both receptor states. This result suggests that the success depends on the matching receptor state and, according to this assumption, a partially activated receptor state would improve the docking results and further analysis based on them. The compounds in the in silico studies are referred to by serial numbers instead of chemical names for simplicity. By this reason, the numbering scheme of the newly synthesized compounds in the docking studies also differs from that in the synthetic schemes: **1** = **9b**, 2 = **9a**, 3 = **11b**, 4 = **10a**, 5 = **12c**, 6 = **11a**, 7 = **12a**, 8 = **11c**, 9 = **12b**, 10 = **10c**, 11 = **10b**, 12 = **9c**. The details of the ligands can be found in the supplemental materials.

#### Structure–property relationships: classification by docking energy (PCA)

Although the docking energy differences between the active and inactive states (E_active_ − E_inactive_) did not result in an unambiguous classification of the ligands of different pharmacological types, i.e. agonists, antagonists, partial agonists, there is a clear tendency for the separation (Supplementary Fig. S8).

Therefore, the results were further analyzed with the PCA (Principal Component Analysis) method. First biplot PCA was used (Supplementary Fig. S9) with overlapping fields as result. However, using the docking energy differences, ligand efficiency differences and the docking pose dependent ligand efficiency differences, a PCA (Principal Component Analysis) classification resulted in a better separation of agonists and antagonists shown by the distance matrix (Supplementary Fig. S10).

**Figure 3:**
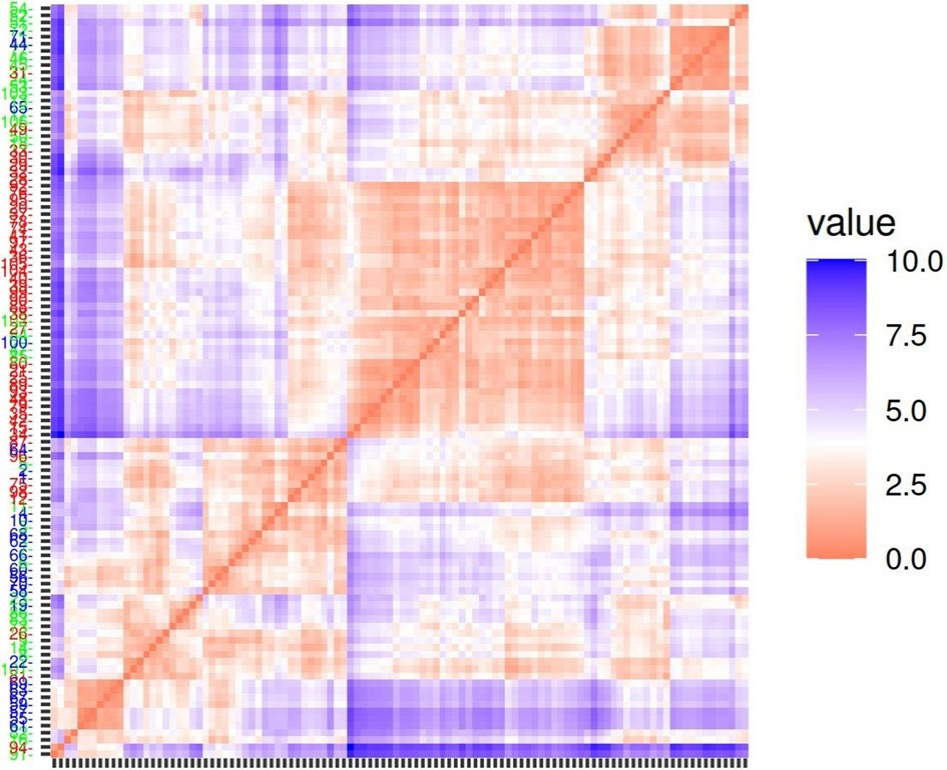
Distance matrix based on docking energies and their differences. Results obtained with five PCA variables using the docking energies, ligand efficiencies, the docking pose-dependent ligand efficiencies and their differences obtained for the active and inactive receptor states. Numbering scheme of the newly synthesized compounds in the docking results: 1 = **9b**, 2 = **9a**, 3 = **11b**, 4 = **10a**, 5 = **12c**, 6 = **11a**, 7 = **12a**, 8 = **11c**, 9 = **12b**, 10 = **10c**, 11 = **10b**, 12 = **9c**.

The separation of agonists and antagonists was quite efficient, although partial agonists were mainly mixed with antagonists. Further improvement was achieved by using not only the differences of the docking energy measures but also their values as shown by the distance matrix obtained by PCA analysis with five components (Fig. 3). Although there are overlapping ligands in all groups, the separation of the ligands of different characters is unequivocal. It is noteworthy that although only the active and inactive receptor states were used, the three pharmacological types were separated in three major groups.

#### Structure–function relationships: classification by interacting receptor atoms using multiple correspondence analysis (MCA)

The behavior of the receptor when it binds a ligand is an obvious consequence of the specific interactions formed. The forces resulting from the interactions will shift and/or stabilize the receptor to different conformations (states) responsible for the particular pharmacological responses. According to this, the pharmacological effect of the ligands is reflected on the receptor side, i.e. by the set of interactions the receptor observes. In the present paper, a simple description is used, namely the interactions are only rated by their stabilizing or destabilizing nature, determined for each interacting receptor atom. This resulted in a binary data table for each ligand. Merging the data tables of all ligands, being investigated together, serves the input for multivariate classification.

**Figure 4:**
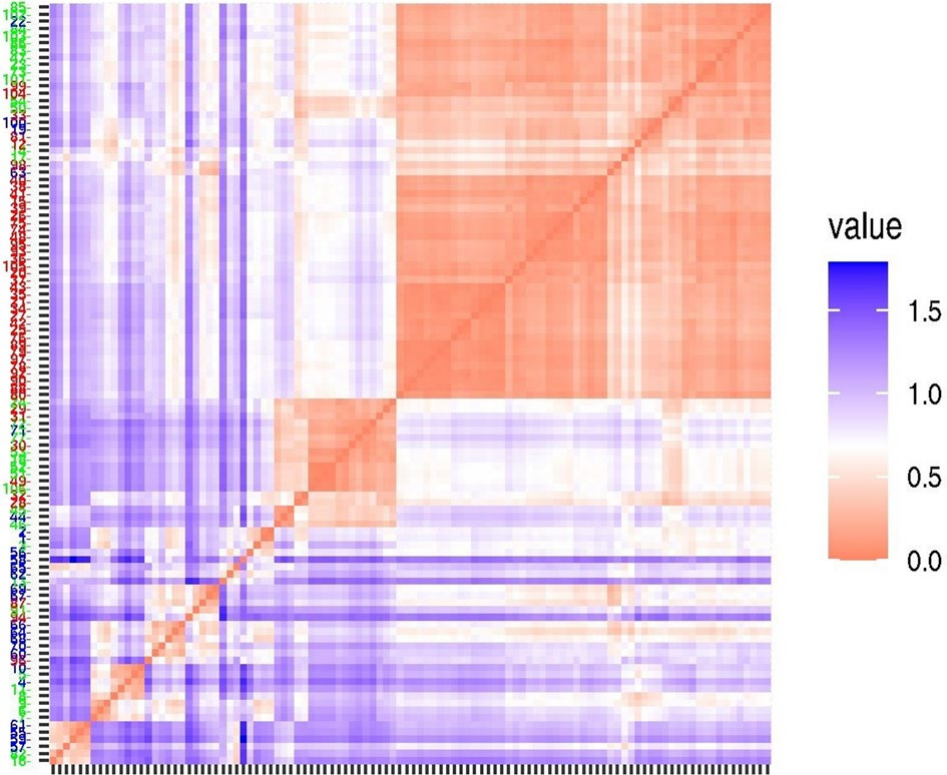
Distance matrix of multiple correspondence analysis results. Results obtained with five MCA variables. Numbering scheme of the newly synthesized compounds in the docking results: 1 = **9b**, 2 = **9a**, 3 = **11b**, 4 = **10a**, 5 = **12c**, 6 = **11a**, 7 = **12a**, 8 = **11c**, 9 = **12b**, 10 = **10c**, 11 = **10b**, 12 = **9c**.

Using this binary input, the classification of the ligands by MCA resulted in a significant overlap between the pharmacological groups relying only on the first two dimensions of the MCA results (Supplementary Fig. S11). The maximal efficiency of MCA can be seen on the distance matrix where all five dimensions are considered (Fig. 4). As it is seen, yet there are overlaps among the pharmacological features, i.e. mixed groups exist but the compounds can definitely be separated by their pharmacological features.

#### Classification by hierarchical clustering

Further improvement of the pharmacological grouping was assumed using clustering methods on the principal components (factors) obtained by MCA. Clustering was performed by HCPC (Hierarchical Clustering on Principal Components) which uses an agglomerative hierarchical clustering algorithm. The efficiency of hierarchical clustering is demonstrated by the distance matrix of the interactions involving five principal components (Supplementary Fig. S12). Most of the agonists were classified in one major group while antagonists and partial agonists formed several smaller groups. Although mixed pharmacological groups also exist, mostly partial agonists are mixed to the other two types. This may be a consequence of the absence of the partial agonist state receptor model for which partial agonists would bind by a particular manner compared to the other two types. Compounds **1** and **2** (partial agonists) as well as compound **3** (antagonist) belong to a bigger group of partial agonists despite numerous differences in their structures. Compounds **4–11** (antagonists and partial agonists) were grouped together, although the grouping was less tight. It is noteworthy that these compounds have numerous variations in their structures including the ring size and composition and amidation of the ring N-atom in the 17-N- substituent and absence or presence of the 14-OH group. Compound **12**, the only full agonist among the new compounds is in a bigger group composed of mainly partial agonists.

## Discussion

### In vitro

The three-way variation of the functional groups in the new compounds (i: ring of the 17-N-substituent; ii: amide formation with the ring nitrogen; iii: presence or absence of 14-OH and 6-oxo in the morphinan skeleton) prevents the straightforward estimation of the binding and pharmacological properties of the ligands because the effects of the substituents is not additive. However, a pairwise comparison of the experimental data can still reveal some trends in both the binding affinity values and the efficacy. According to the data in Tables 1 and 2. (i) Compounds with piperidine substitution at position 17 and carbonyl at position 19 did not bind to any opioid receptors or showed very low affinity. (ii) Acylation of the ring nitrogen resulted in decreased binding affinity compared to compounds without acylation. (iii) All noroxymorphone (**11a**, **11b**, **11c**, **12a**, **12b**, **12c**) analogues which had OH at 14 position, showed antagonism, while normorphine analogues (**9a**, **9b**, **9c**, **10a**, **10b**, **10c**) which did not have OH at 14 position, showed agonism or partial agonism with higher affinity towards KOR than to MOR and DOR.

### In silico

Considering the presence or absence of particular functional groups in the newly synthesized compounds, their affinities (K_i_) and pharmacological profiles (agonist, antagonist, partial agonist) could not be estimated in a straightforward manner. A feasible explanation of this fact is that binding one part of the ligand to the receptor can affect the binding of other parts i.e. the effect of the functional groups would not be exactly additive. However, the hypothesis that the interactions received by the receptor from the ligands unambiguously determine the response of the receptor, implies the idea that determining these interactions would allow the estimation of the type of the response, i.e. the pharmacological profiles of the ligands. Identification of the receptor-ligand interactions was performed by molecular docking, and they were classified as stabilizing or destabilizing by nature. Using this kind of description of the receptor-ligand interactions, a combined multiple correspondence analysis and hierarchical clustering was partially able to classify the ligands by their pharmacological profile in an unsupervised manner. Although the method needs improvement, this simplified description of the receptor-ligand interactions can account for the pharmacological profiles of the ligands efficiently, using the fast molecular docking method and multivariate statistical analysis. Similarly, the docking energy-based method is also able to differentiate the ligands of different pharmacological types involving both the docking energy measures and their difference calculated for the distinct receptor states.

## Materials and methods

### Chemistry

#### General information

The reagents and indicator molecules were purchased from Sigma-Aldrich and Alfa Aesar and used without further purification. Solvents were freshly distilled prior to use and were dried over anhydrous Na_2_SO_4_. ^1^H and ^13^C NMR spectra were recorded on a 600-MHz Varian VNMRS spectrometer in DMSO-d_6_ or chloroform-d_1_ solutions; δ is given in ppm relative to TMS as internal standard. ^1^H and ^13^C NMR signals were assigned by one- and two-dimensional homo- and heteronuclear experiments (HMBC and HSQC). Melting points were taken on a Stuart SMP-3 apparatus. The high-resolution accurate masses were determined with a Dionex Ultimate 3000 UHPLC system hyphenated with a Orbitrap Q Exactive Focus Mass Spectrometer equipped with electrospray ionization (ESI) (Thermo Fischer Scientific, Waltham, MA, USA) was used for high resolution mass spectral analysis. Reaction progress was observed by thin-layer chromatography on commercial silica gel plates (Merck Kieselgel 60 silica gel F254 on aluminum sheets) using different mobile phases. For column chromatography, Kieselgel 60 (particle size 0.040–0.063 mm) was employed.

**Figure 5:**
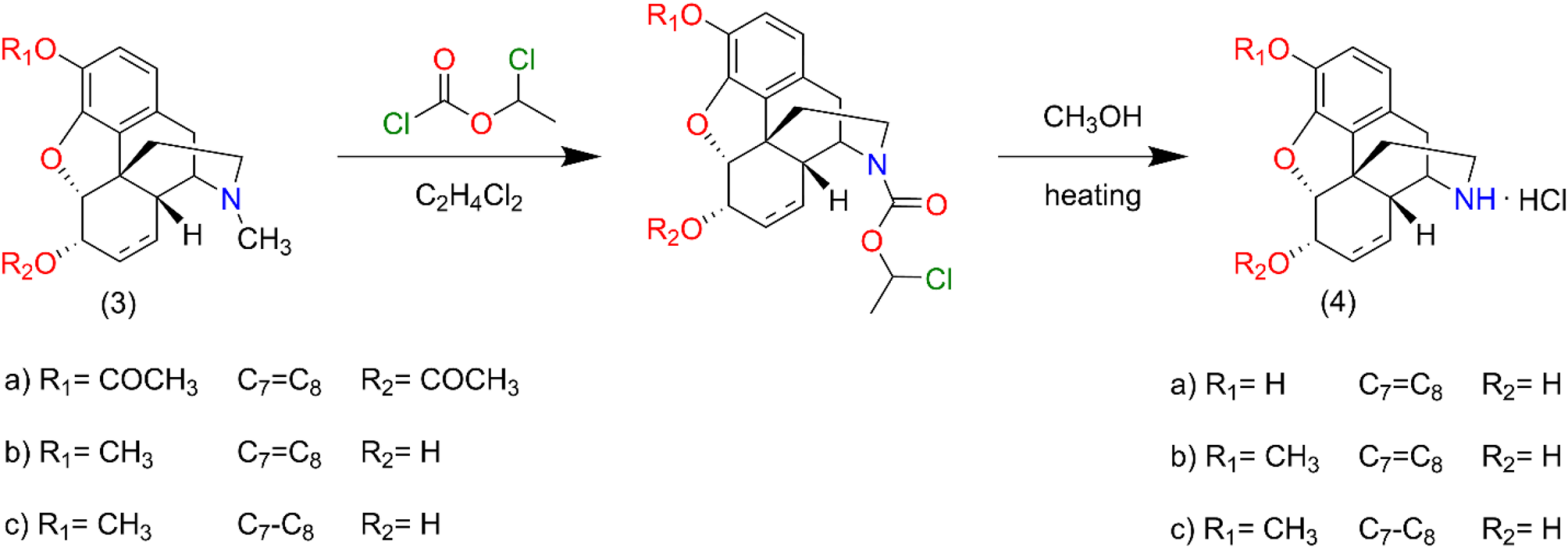
Synthesis route for normorphine derivatives. Preparation of norcodeine (**4b**), dihydronorcodeine (**4c**) and normorphine (**4a**).

Preparation of normorphinans was accomplished with the method of Olofson^32^: codeine (**3b**) or dihydrocodeine (**3c**) were treated with α-chloro-ethyl chloroformate in 1,2-dichloroethane solvent and the intermediate carbamate was decomposed in methanol at 50 °C to yield the hydrochloride salt of norcodeine (**4b**) (dihydronorcodeine **4c**). The 17-N-demethylation of 3,6-diacetyl morphine (**3a**) with α-chloroethyl chloroformate yielded the hydrochloride salt of 3,6-diacetyl normorphine which was hydrolyzed with 5% HCl at 100 °C to obtain **4a** (Fig. 5).

**Figure 6:**
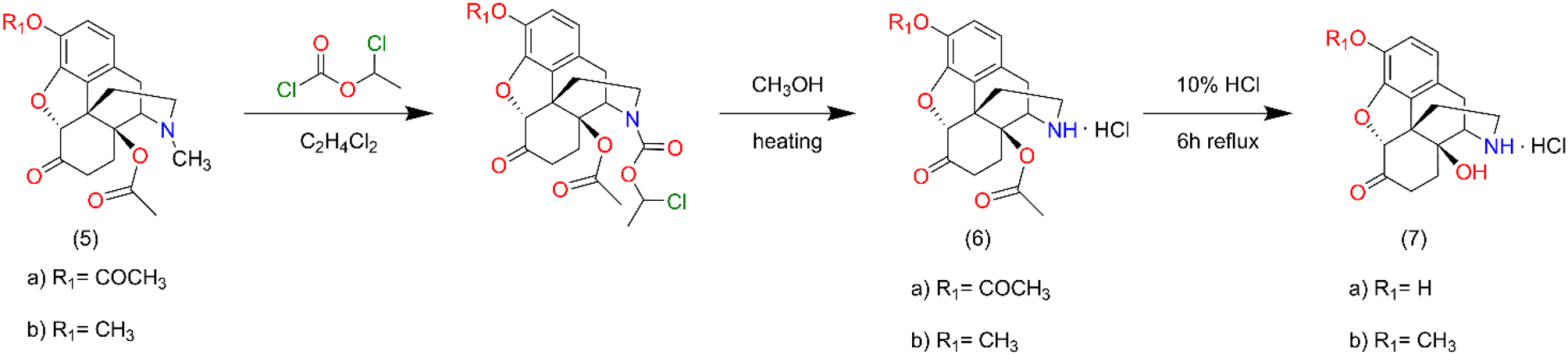
Synthesis route for noroxycodone and noroxymorphone series. Preparation of noroxycodone (**7b**) and noroxymorphone (**7a**).

For oxycodone the same procedure was utilized starting from 14-O-acetyloxycodone (**5b**) but to avoid O-N acyl migration the product 14-O-acetylnoroxycodone hydrochloride salt (**6b**) was hydrolyzed with 10% HCl at reflux temperature for 16 h to obtain noroxycodone (**7b**). **7a** was prepared from 3,14-diacetyl-oxymorphone (**5a**) in similar reactions (Fig. 6).

**Figure 7:**
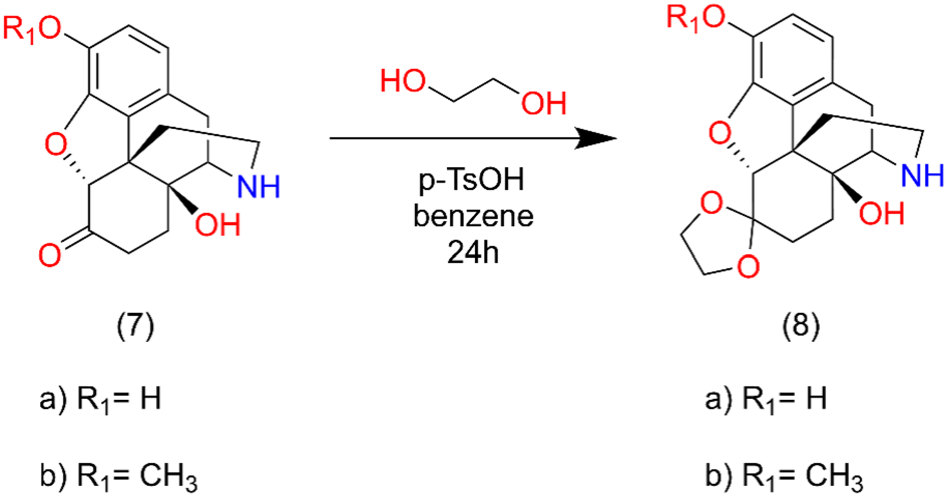
Synthesis of ethylene ketal intermediates.

The C-6 keto group of noroxycodone and noroxymorphone was protected by ethylene ketal formation (**7b → 8b** and **7a → 8a**) in order to increase the solubility of normorphinans. After N-alkylation the ketal protecting group was removed by acid hydrolysis (Fig. 7).

The 4,5-epoxynormorphinans were N-alkylated with β-chloroethylamine (**9a-i**, **11a-f**) in methanol in the presence of sodium hydrogen carbonate at 50 °C. The chloroacetamides were prepared by acylation of secondary amines (morpholine, piperidine and pyrrolidine) with chloroacetyl chloride in dichloromethane or tetrahydrofuran. The N-alkylation of 4,5-epoxy-normorphinans with chloroacetamides (**10a-i**, **12a-f**) was achieved in dimethyl formamide using sodium hydrogen carbonate as acid scavenger. The structures of the new derivatives were elucidated by analysis of the NMR and MS spectra.

Codeine (**3b**) or dihydrocodeine (**3c**) (3 mmol) was dissolved in dried 1,2-dichloroethane (30 mL) then sodium hydrogen carbonate (0.84 g) was added to the solution. To this ice-cold mixture α-chloroethyl chloroformate (10 mmol) was added dropwise and the reaction mixture was stirred at room temperature for 30 min. and then heated at 90 °C overnight. The resulting suspension was cooled to room temperature and the inorganic salts were filtered off. The filtrate was evaporated under reduced pressure, the residue was dissolved in methanol and the solution was heated at 60 °C for 6 h. Methanol was removed under reduced pressure to yield a crystalline solid, the hydrochloride salt of norcodeine or dihydronorcodeine. The free base was liberated with 10% sodium hydroxide (pH = 9) and was extracted with chloroform. The chloroform extract was dried (sodium sulphate) and evaporated to result in the norcompound. Norcodeine yield: 87%, m.p.: 185 °C (acetone). Dihydronorcodeine yield: 83%, m.p.: 194 °C (ethanol).

Normorphine was prepared by 17-N-demethylation of diacetyl morphine (**3a**) by means of α-chloro-ethyl chloroformate using the above-mentioned procedure and the carbamate was treated with methanol at 60 °C to afford diacetyl normorphine hydrochloride salt. Normorphine was obtained by hydrolysis of diacetyl normorphine x HCl in 5%HCl at 100 °C for 4 h. Normorphine base was precipitated with 25% ammonia solution (pH = 9–10) and filtered off. Yield: 84%, m.p.: 273–275 °C.

#### Preparation of noroxycodone (**7b**) and noroxymorphone (**7a**)

14-O-acetyloxycodone (**5b**) (2 mmol) was 17-N-demethylated with α-chloroethyl chloroformate (10 mmol) for 16 h. The reaction was monitored by thin-layer chromatography to check the conversion, if it was necessary another 5 mmol portion of α-chloro-ethyl chloroformate was added. The carbamate intermediate was decomposed in methanol to yield the hydrochloride salt of 14-O-acetylnoroxycodone. Acid hydrolysis in refluxing 10% HCl for 6 h resulted in the hydrochloride salt of noroxycodone. The free base of noroxycodone was liberated from the acid solution 10% sodium hydroxide (pH = 10) and it was extracted with chloroform. The chloroform extract was dried under sodium sulphate and evaporation of the solution afforded the oily noroxycodone, which was rubbed with diethyl ether to produce crystalline material. Yield: 86%, m.p.: 163–166 °C.

17-N-demethylation of 3,14-di-O-acetyloxymorphone (**5a**) yielded the hydrochloride salt of 3,14-di-O-acetylnoroxymorphone, which was hydrolyzed in 10% HCl solution. Noroxymorphone base was precipitated from the acid solution with 25% ammonia solution to yield noroxymorphone which was pure for further reactions. Yield: 81% m.p.: > 280 °C.

A mixture of 5.0 g of noroxycodone (**7b**), 10 mL of ethylene glycol, 4.0 g of p-toluenesulfonic acid and 60 mL of toluene was boiled and stirred under reflux for 24 h. The separated water was removed using a Dean-Stark trap. The reaction mixture was cooled to room temperature and toluene was removed in vacuum. The residue was dissolved in water and the product was precipitated under cooling with 20% sodium hydroxide adjusting the pH to 12. Noroxycodone ethylene ketal was filtered off and washed with cold water. Yield: 4.4 g and 81% m.p.: 197–199 °C after recrystallization from benzene.

Noroxymorphone ethylene ketal (**8a**) was prepared by the same procedure, starting from 3,0 g of (**7a**), 10 mL of ethylene glycol, 2.5 g of p-toluenesulfonic acid and 60 mL of toluene. Noroxymorphone ethylene ketal was precipitated from the aqueous solution by 25% ammonia solution. After filtration 2.5 g of ketal was obtained. M.p.: > 305 °C (decomp.).

#### Hydrolysis of ethylene ketals

Heating 2.0 g of ketal with 30 mL of 5% hydrochloric acid for 3 h on a steam bath resulted in hydrolysis of the protecting group. The solution was cooled to room temperature and the pH was adjusted to 9 with 25% ammonia solution and the product was isolated by chloroform extraction.

**Figure 8:**
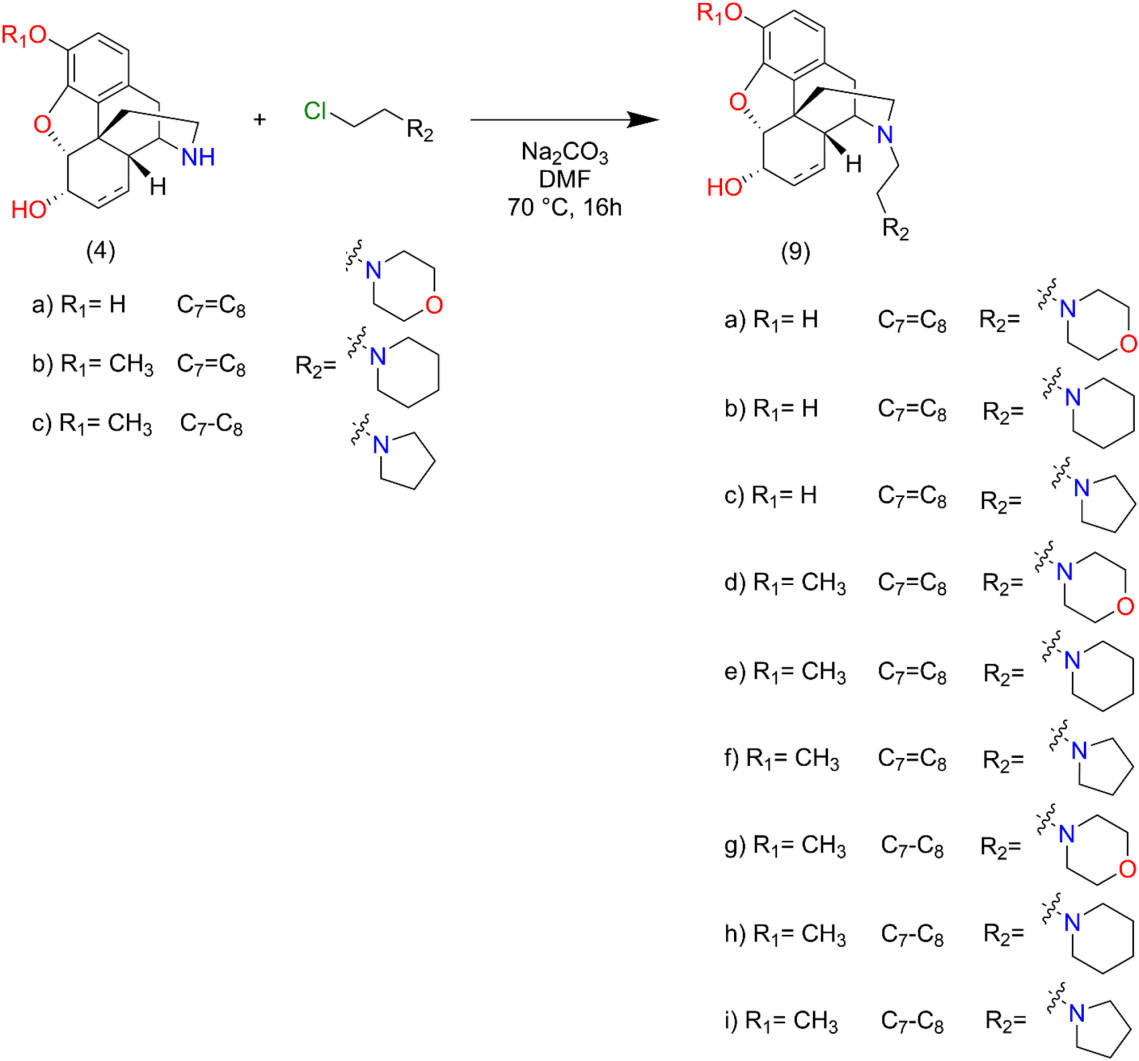
17-N-Alkylation of normorphinans (normorphine, norcodeine, dihydronorcodein) with β-chloroethylamines.

Normorphinan (3 mmol), β-chloroethylamine hydrochloride (4.2 mmol), sodium carbonate (1.27 g, 12 mmol) and a catalytic amount of KI was dissolved in dry dimethyl formamide, and the reaction mixture was stirred and heated at 70 °C for 16 h. The reaction mixture was cooled to room temperature and the inorganic salts were filtered off. The filtrate was evaporated in vacuo, the residue was dissolved in water and 25% ammonia solution was added to adjust the pH to 9. The product was isolated by chloroform extraction, the chloroform extract was washed with brine and dried over sodium sulphate. After evaporation the crude products were purified by silica gel column chromatography, using chloroform–methanol 9:1 (v/v) eluent. Detailed NMR and MS spectra figures of the target compounds **9a**, **9b** and **9c** can be found in Supplementary Figs. S13, S14 and S15, respectively (Fig. 8).

**Figure 9:**
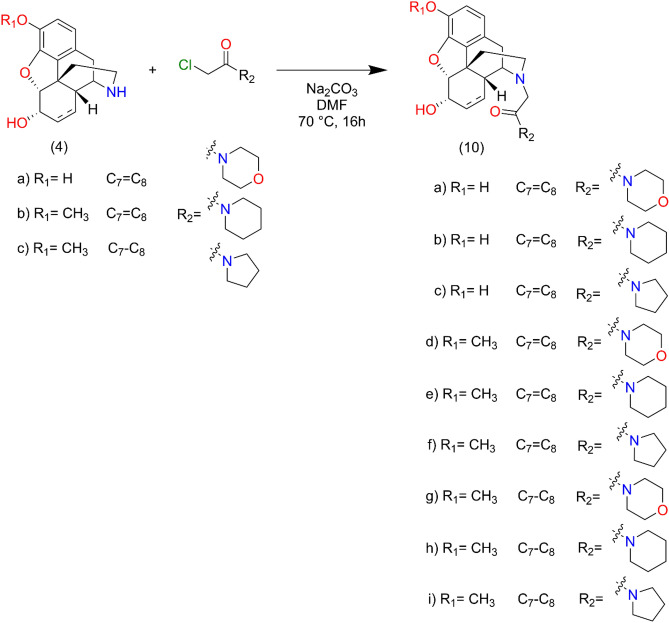
Synthesis route of 17-N amide derivatives.

Normorphinan (3 mmol), N-chloroacetyl piperidine (pyrrolidine, morpholine) (3,7 mmol), sodium carbonate (1.27 g, 12 mmol), and a catalytic amount of KI was dissolved in dry dimethyl formamide, and the reaction mixture was stirred and heated at 70 °C for 16 h. The reaction mixture was cooled to room temperature and the inorganic salts were filtered off. The filtrate was evaporated in vacuo, and the residue was dissolved in water and 25% ammonia solution was added to adjust the pH to 9. The product was isolated by chloroform extraction, the chloroform extract was washed with brine and dried over sodium sulphate. After evaporation the crude products were purified by silica gel column chromatography, using chloroform–methanol 9:1 (v/v) eluent. Detailed NMR and MS spectra figures of the target compounds **10a**, **10b** and **10c** can be found in Supplementary Figs. S16, S17 and S18, respectively (Fig. 9).

**Figure 10:**
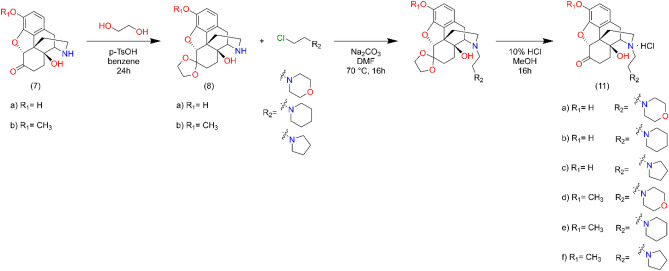
N-Alkylation of noroxymorphone and noroxycodone.

Normorphinan (3 mmol) β-chloroethylamine hydrochloride (4.2 mmol), sodium carbonate (1.27 g, 12 mmol), and a catalytic amount of KI was dissolved in dry dimethyl formamide, and the reaction mixture was stirred and heated at 70 °C for 16 h. The reaction mixture was cooled to room temperature and inorganic salts were filtered off. The filtrate was evaporated in vacuo, and the residue was dissolved in water and 25% ammonia solution was added to adjust pH to 9. The product was isolated by chloroform extraction; the chloroform extract was washed with brine and dried over sodium sulphate. After evaporation the crude products were purified by silica gel column chromatography, using chloroform–methanol 9:1 (v/v) eluent. Ketal protecting groups were hydrolyzed in 5% HCl. Detailed NMR and MS spectra figures of the target compounds **11a**, **11b** and **11c** can be found in Supplementary Figs. S19, S20 and S21, respectively (Fig. 10).

**Figure 11:**
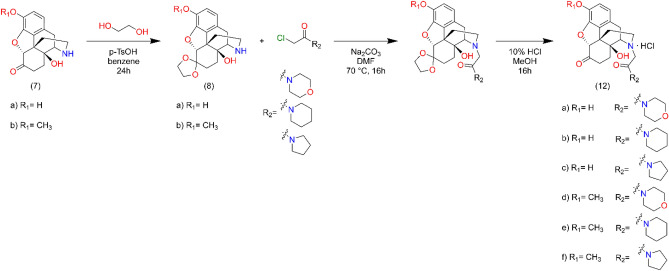
Synthesis route of 17-N substituted amide derivatives of noroxymorphone and noroxycodone.

Normorphinan (3 mmol), N-chloroacetylpiperidine (pyrrolidine, morpholine) (3,7 mmol), sodium carbonate (1.27 g, 12 mmol), and a catalytic amount of KI was dissolved in dry dimethyl formamide, and the reaction mixture was stirred and heated at 70 °C for 16 h. The reaction mixture was cooled to room temperature and inorganic salts were filtered off. The filtrate was evaporated in vacuo, and the residue was dissolved in water and 25% ammonia solution was added to adjust pH to 9. The product was isolated by chloroform extraction, the chloroform extract was washed with brine and dried over sodium sulphate. After evaporation the crude products were purified by silica gel column chromatography, using chloroform–methanol 9:1 (v/v) eluent. Ketal protecting groups were hydrolyzed in 5% HCl. Detailed NMR and MS spectra figures of the target compounds **12a**, **12b** and **12c** can be found in Supplementary Figs. S22, S23 and S24, respectively (Fig. 11).

The NMR data of the intermediate compounds can be found in the Supplementary material after those of the target compounds.

### In vitro experiments

#### Chemicals

Tris–HCl (tris-(hydroxymethyl)-aminomethane hydrochloride), EGTA (ethyleneglycol-tetraacetate), NaCl, MgCl_2_ × 6H_2_O, GDP (guanosine diphosphate), the GTP (guanosine 5′-triphosphate) analogue GTPγS (guanosine-5′-O-(3-thiotriphosphate)) and KOR selective agonist U-69,593 were purchased from Sigma-Aldrich (Budapest, Hungary). The highly selective MOR agonist enkephalin analogue, DAMGO was obtained from Bachem Holding AG (Bubendorf, Switzerland), the MOR agonist morphine and **4a** were kindly provided by Dr. Hosztafi Sándor. The highly selective DOR agonist, Ile^5,6^-deltorphin II was purchased from Isotope Laboratory of BRC (Szeged, Hungary). The selective MOR antagonist cyprodime and the highly selective KOR agonist diphenethylamine derivative, HS665^33^ were prepared by Dr. Helmut Schmidhammer (University of Innsbruck, Austria). The non-selective opioid receptor antagonist naloxone was kindly provided by the company Endo Laboratories DuPont de Nemours (Wilmington, DE, USA). The selective DOR antagonist NTI and the selective KOR antagonist nor-BNI were obtained from Tocris Bioscience (Bristol, UK). DAMGO, morphine and Ile^5,6^-deltorphin II were dissolved in water, morphine analogues were dissolved in ethanol and were stored in 1 mM stock solution at − 20 °C. The radiolabeled GTP analogue, [^35^S]GTPγS (specific activity: 1000 Ci/mmol) was purchased from Hartmann Analytic (Braunschweig, Germany). [^3^H]DAMGO (specific activity: 38.8 Ci/mmol)^34^, [^3^H]Ile^5,6^-deltorphin II (specific activity: 19.6 Ci/mmol) and [^3^H]HS665 (specific activity: 13.1 Ci/mmol) were radiolabeled in the Isotope Laboratory of BRC (Szeged, Hungary)^35^. The UltimaGold™ MV aqueous scintillation cocktail was purchased from PerkinElmer (Boston, USA).

#### Animals

Both male and female Wistar rats and guinea pigs were used for the membrane preparations. The rats were 10-week-old weighing 250–300 g, the guinea pigs were 8-week-old weighing 450–500 g. The animals were kept in a temperature-controlled room (21–24 °C) under a 12:12 light and dark cycle and were provided with water and food ad libitum. All housing and experimental conditions were performed in accordance with the European Communities Council Directives (2010/63/EU) and the Hungarian Act for the Protection of Animals in Research (XXVIII.tv. 32.§). These conditions also comply with the ARRIVE guidelines (https://arriveguidelines.org).

#### Preparation of brain samples for binding assays

The animals were anesthetized with isoflurane and killed by rapid decapitation, then their brains were quickly removed. The brains were prepared for membrane preparation according to Benyhe^36^ and partly used for binding experiments, and partly were further prepared for the [^35^S]GTPγS binding experiments according to Zádor^37^.

The brains were homogenized in 30 volumes (v/w) of ice-cold 50 mM Tris–HCl pH 7.4 buffer with a Teflon-glass Braun homogenizer operating at 1500 rpm. The homogenate was centrifuged at 18,000 rpm for 20 min at 4 °C, the resulting supernatant discarded, and the pellet taken up in the original volume of Tris–HCl buffer. The homogenate was incubated at 37 °C for 30 min in a shaking water-bath. Then centrifugation was repeated as described before. The final pellet was resuspended in 5 volumes of 50 mM Tris–HCl pH 7.4 buffer, stored at − 80 °C.

For the [^35^S]GTPγS binding experiments the brains were homogenized with a Dounce in 5 volumes (v/w) of ice-cold TEM (Tris–HCl, EGTA, MgCl_2_) and stored at − 80 °C. The protein content of the membrane preparation was determined by the method of Bradford, BSA being used as a standard^38^.

### Receptor binding assays

#### Binding experiments

In MOR, DOR and KOR displacement aliquots of frozen rat and guinea pig brain membrane homogenates were thawed and suspended in 50 mM Tris–HCl buffer (pH 7.4). Membranes were incubated in the presence of the unlabeled ligands in increasing concentrations (10^–10^–10^–5^ M) at 35 °C for 45 min with [^3^H]DAMGO, at 35 °C for 45 min with [^3^H]Ile^5,6^-deltorphin II and at 25 °C for 30 min with [^3^H]HS665. The non-specific and total binding were determined in the presence and absence of 10 µM unlabeled naloxone (MOR and DOR) and HS665 (KOR). The reaction was terminated by rapid filtration under vacuum (Brandel M24R Cell Harvester) and washed three times with 5 mL ice-cold 50 mM Tris–HCl (pH 7.4) buffer through Whatman GF/C glass fibers. The radioactivity of the dried filters was detected in UltimaGold™ MV aqueous scintillation cocktail with Packard Tricarb 2300TR liquid scintillation counter. The competitive binding assays were performed in duplicates and repeated at least three times.

#### Functional [^35^S]GTPγS binding experiments

The functional [^35^S]GTPγS binding experiments were performed as described previously with modifications^39,40^. Briefly the membrane homogenates were incubated at 30°C for 60 min in Tris-EGTA buffer (pH 7.4) composed of 50 mM Tris–HCl, 1 mM EGTA, 3 mM MgCl_2_, 100 mM NaCl, containing 20 MBq/0.05 cm^3^ [^35^S]GTPγS (0.05 nM) and increasing concentrations (10^–10^–10^–5^ M) of ligands. The experiments were performed in the presence of excess GDP (30 µM) in a final volume of 1 mL. Total binding was measured in the absence of test compounds, non-specific binding was determined in the presence of 10 µM unlabeled GTPγS and subtracted from total binding. The difference represents basal activity. The reaction was terminated by rapid filtration under vacuum (Brandel M24R Cell Harvester) and washed three times with 5 mL ice-cold 50 mM Tris–HCl (pH 7.4) buffer through Whatman GF/B glass fibers. The radioactivity of the dried filters was detected in UltimaGold™ MV aqueous scintillation cocktail with Packard Tricarb 2300TR liquid scintillation counter. [^35^S]GTPγS binding experiments were performed in triplicates and repeated at least three times.

### Data analysis

Experimental data were presented as means ± S.E.M. Points were fitted with the professional curve fitting program, GraphPad Prism 5.0 (GraphPad Prism Software Inc., San Diego, CA), using non-linear regression. In the competition binding assays, the ‘One site competition’ fitting was used to establish the equilibrium binding affinity (K_i_ value). Inhibition was given as percent of the specific binding observed.

In the [^35^S]GTPγS binding assays the ‘Sigmoidal dose–response’ fitting was used to establish the maximal stimulation or efficacy (Emax) of the receptors G-protein and the ligand potency (EC_50_). Stimulation was given as percent of the specific [^35^S]GTPγS binding observed over basal activity, which was settled as 100%. Unpaired t-test with two-tailed P-value was performed to determine the significance level using GraphPad Prism 5.0. Significance was accepted at the P < 0.05 level.

### In silico calculations

Pharmacological profiling of the new compounds was performed by unsupervised classification of a set of compounds including the new compounds and others with known pharmacological features. The compounds were docked to the active and inactive states of MOR and the docked poses were clustered by multivariate statistical methods to group the compounds by their pharmacological feature.

Docking calculations: the ligands were flexibly docked to rigid receptor models using the experimental structures of both the active and inactive states. Docking was performed by the docking program PLANTS with the following additional parameters^41^: chemplp_protein_hb_constraint = 3 for the ASP147 carboxyl oxygen atoms, chemplp_charged_hb_weight = 3, ligand_intra_score = lj, chemplp_clash_include_HH = 1. The first two parameters were applied to force the docking algorithm to find the charged interaction between the ASP147 side chain and the 17-N of the morphinan skeleton. Preparation of the receptor for docking was performed by SPORES^42^. Receptor models: 3D coordinates of the active and active states were downloaded from https://www.rcsb.org/, PDB codes are 5C1M and 4DKL, respectively. Ligand preparation: 3D structures were drawn using Avogadro^43^. Substituents on the 17N of the morphinan skeleton were equatorial. All compounds were protonated to pH 7.4. The structures were energy minimized by Open Babel^44^, using MMFF94s force field and conjugated gradient minimization strategy with criteria of 0.0001 kcal/mol and 100,000 for energy gradient and maximum number of iterations, respectively. Individual docking sphere radius was determined by SPORES for each ligand and then increased by 5 Angstroms to perform docking. Docking was repeated five times and the docking poses with lowest docking energy were selected for further investigations^31^, involving both the docking energies and the interacting receptor atoms specific for each selected pose. Both types of data were subjected to unsupervised classification together with a set of ligands with known pharmacological features.

Multivariate analysis of the docking energies: Instead of using the scores resulted by PLANTS, the docked poses were rescored by PSOVina^45^, which estimates a binding affinity in kcal/mol. Prior to this the docked poses by PLANTS were transformed into PDBQT format by AutoDock Tools^46^. Different interaction energy measures were used, namely the docking energy calculated, the ligand efficiency (docking energy divided by the number of the non-hydrogen atoms of the ligand) as well as a modified ligand efficiency value (docking energy divided by the number of the interacting receptor atoms). Furthermore, the difference between these energy measures were also used by subtracting the value obtained for the inactive receptor from the value obtained for the active receptor. The input data were processed by the PCA module of the FactoMineR package^47^ of R^48^ with 5 principal components. Results were visualized by Factoextra^49^ and ggplot2^50^.

Multivariate statistical analysis of interacting receptor atoms: interacting atoms in the receptor-ligand complexes were obtained by the docking pose analyzer program BINANA^51^. The type of the interactions (i.e. stabilizing or destabilizing) was determined by the conformity of the interacting atom types (polar-polar, polar-apolar, etc.)^31^, and the binary table of the classified interactions of the receptor atoms served the input for the MCA module of the FactoMineR package of R. The classification by MCA was further refined by agglomerative hierarchical clustering performed on the resulting MCA factors by the modules HCPC and FactoInvestigate^52^ of FactoMineR.

Molecular structures in svg format were drawn by Bkchem v0.13.0 and the final graphics files were obtained by Inkscape v0.91 and ImageMagick v6.7.7.

### Ethics declarations

Housing and experiments with rats and guinea pigs were conducted in accordance with the European Communities Council Directives (86/606/ECC) and the Hungarian Act for the Protection of Animals in Research (XXVIII.tv. 32.§). The total number of animals as well as their suffering was minimized whenever possible.

### Animal experimentation and ethics declaration

All experimental procedures involving animals were reported in accordance with ARRIVE guidelines for reporting animal experiments in addition to the compliance with the European Communities Council Directives (2010/63/EU) and the Hungarian Act for the Protection of Animals in Research (XXVIII.tv. 32.§), in possession of the approval of Institutional Animal Experimentation and Ethics Committee of the Biological Research Centre (SZBK MÁB).

### Supplementary Information

Supplementary Information.

## Data Availability

Data generated by docking calculations, both the energies and the patterns of the interacting receptor atoms are available in the Supplementary materials.
