# Supplementary material for: Synthesis and biochemical evaluation of 17-N-beta-aminoalkyl-4,5α-epoxynormorphinans
Source: Sci Rep. 2023 Nov 20;13:20305. doi: 10.1038/s41598-023-46317-3 (PMC10660610; doi:10.1038/s41598-023-46317-3)
Supplement: Supplementary file 1 — Supplementary Information. [file 41598_2023_46317_MOESM1_ESM.zip › Supplement_scirep4.docx]

**Supporting Information**

**Synthesis and Biochemical Evaluation of 17-*N*-beta-Aminoalkyl-4,5α-Epoxynormorphinans**

Ferenc Ötvös^*,+,1^, Edina Szűcs^+,1^, Ákos Urai^+,2^, István Köteles^2^, Pál T. Szabó^3^, Zsuzsanna Katalin Varga^1,4^, Dávid Gombos^1,4^, Sándor Hosztafi^2^ and Sándor Benyhe^*,1^

^1^ Institute of Biochemistry, Biological Research Centre, Temesvári krt. 62, H-6726 Szeged, Hungary

^2^ Institute of Pharmaceutical Chemistry, Semmelweis Medical University, Hőgyes Endre utca 9, H-1092, Budapest, Hungary

^3^ Research Centre for Natural Sciences, MS Metabolomics Research Laboratory, H-1117, Budapest, Magyar tudósok krt. 2, Hungary

^4^ Theoretical Medical Doctoral School, Faculty of Medicine, University of Szeged, H-6726 Szeged, Hungary

^+^ these authors contributed equally to this work

***** otvos@brc.hu, benyhe@brc.hu

*Address correspondence to Dr. Ferenc Ötvös and Sándor Benyhe: Institute of Biochemistry, Biological Research Center, Temesvári krt. 62, H-6726 Szeged, Hungary; e-mail: otvos@brc.hu and benyhe@brc.hu

**Table of Contents**

Cover	page 1

Contents	page 2

In vitro binding curves	page 3

In silico docking and clustering supplementary figures	page 7

Chemical Synthesis and analysis supplementary information	page 9

Target Compound NMR and Mass spectrometry results	page 9

Other Compounds NMR spectrum results	page 33

Supplemental information related to the docked ligands	page 40

### In vitro binding curves

**Figure S1**. Binding affinities of the noroxymorphone (**7a**) and normorphine (**4a**) analogues.

Binding affinities for MOR (A, B), DOR (C, D) and KOR (E, F) were determined by competition binding assays in rat and guinea pig brain membrane homogenates with [^3^H]DAMGO, [^3^H]Ile^5,6^-deltorphin II and [^3^H]HS665, respectively. Membranes were incubated with 2 nM radioligand. Values represent mean values ± S.E.M. for at least three experiments performed in duplicate.

**Figure S2**. The effect of the ligands on G-protein activity compared to parent compounds in [^35^S]GTPγS binding assay in rat brain membrane homogenates.

The specific binding of [^35^S]GTPγS was calculated in percentage, in the presence of increasing concentrations (10^-10^ - 10^-5^ M) of the indicated ligands. ‘Total’ on the x-axis indicates the basal activity of the monitored G-protein measured in the absence of the ligands. Basal activity was regarded as 100% (dotted line). Points represent means ± S.E.M. for at least three experiments performed in triplicates.

**Figure S3**. The effect of the ligands on G-protein activity in the absence or presence of the selective opioid agonist and antagonists in [^35^S]GTPγS binding assays.

The effect of noroxymorphone (**7a**) derivatives in the absence or presence of the selective MOR agonist DAMGO and the selective DOR agonist Ile^5,6^-deltorphin II in rat brain membrane homogenates (Figure ESI-BIND 3A) and the selective KOR agonist U-69,593 in guinea pig brain membrane homogenates (Figure ESI-BIND 3B). The effect of normorphine (**4a**) derivatives is shown in the absence or presence of the selective MOR antagonist cyprodime and the selective DOR antagonist NTI in rat brain membrane homogenates (Figure ESI-BIND 3C) and the selective KOR antagonist nor-BNI in guinea pig brain membrane homogenates (Figure ESI-BIND 3D).

The maximal efficacy (E_max_) of the ligands at one concentration (10^-5^ M). The level of basal activity was defined as 100% and it is presented with a dotted line. Points represent means ± S.E.M. for at least three experiments performed in triplicates.

**Figure S4**. The decrease of the effect of the selective agonists by partial agonists in [^35^S]GTPγS binding assays.

**In silico docking and clustering supplementary figures**

**Figure S5**. Docked positions of the target compounds in the active receptor state.

**Figure S6.** Docked positions of the target compounds in the inactive receptor state

**Figure S7.** Distances between the piperidine N of the docked ligands and the ASP147 sidechain of the receptor.

Symbols: agonists circle, antagonist triangle, partial agonists square. Red symbols: active receptor, turquoise symbols: inactive receptor. Distances are in Angstroms.

**Figure S8.** Docking energy differences between the active and inactive receptor states

Red: agonist, green: antagonist, blue: partial agonist. Numbering scheme of the newly synthesized compounds in the docking results: **1**=**9b**, **2**=**9a**, **3**=**11b**, **4**=**10a**, **5**=**12c**, **6**=**11a**, **7**=**12a**, **8**=**11c**. **9**=**12b**, **10**=**10c**, **11**=**10b**, **12**=**9c**.

**Figure S9**. Biplot of the first two PCA vectors calculated for the difference values of the docking energy measures (docking energy; ligand efficiency; docking energy divided by the number of the interacting receptor atoms).

**Figure S10.** Distance matrix based on docking energy differences

Five PCA variables were used for the differences in docking energies, ligand efficiencies and the docking pose-dependent ligand efficiencies obtained for the active and inactive receptor states. Numbering scheme of the newly synthesized compounds in the docking results: **1**=**9b**, **2**=**9a**, **3**=**11b**, **4**=**10a**, **5**=**12c**, **6**=**11a**, **7**=**12a**, **8**=**11c**. **9**=**12b**, **10**=**10c**, **11**=**10b**, **12**=**9c**.

**Figure S11**. The pharmacological groups relying only on the first two dimensions of the MCA results

**Figure S12.** Distance matrix of HCPC results.

Numbering scheme of the newly synthesized compounds in the docking results: **1**=**9b**, **2**=**9a**, **3**=**11b**, **4**=**10a**, **5**=**12c**, **6**=**11a**, **7**=**12a**, **8**=**11c**. **9**=**12b**, **10**=**10c**, **11**=**10b**, **12**=**9c.**

**Synthetical Chemical supplementary information**

**Target Compound NMR and Mass spectrometry results**

**Figure S13.** NMR spectrum of **9a**

**Normorphine-β-*N*-ethylmorpholine (9a, C_22_H_28_N_2_O_4_)**

Yield: 57%

**^1^H NMR (600 MHz, CDCl_3_)** δ 6.63 (d, J = 8.1 Hz, 1H), 6.48 (d, J = 8.1 Hz, 1H), 5.67 (d, J = 9.8 Hz, 1H), 5.32 – 5.23 (m, 1H), 4.86 (d, J = 6.5 Hz, 1H), 4.18 (dq, J = 5.1, 2.4 Hz, 1H), 3.73 (t, J = 4.7 Hz, 4H), 3.44 (dd, J = 6.3, 3.1 Hz, 1H), 2.98 – 2.91 (m, 3H), 2.78 – 2.61 (m, 3H), 2.61 – 2.51 (m, 5H), 2.44 – 2.29 (m, 2H), 2.10 – 1.97 (m, 1H), 1.84 (d, J = 12.6 Hz, 1H), 1.37 (t, J = 7.3 Hz, 3H), 1.26 (d, J = 2.5 Hz, 1H)

**APT NMR (151 MHz, CDCl_3_)** δ 145.39, 139.83, 134.97, 130.91, 127.42, 124.45, 119.81, 113.37, 93.59, 67.10, 66.68, 66.46, 54.61, 53.94, 52.68, 47.03, 45.77, 45.03, 38.82, 21.82

**mp:** 96-97˚C, **MS**: calculated: 385.4766, measured: **HR-MS [M+ H^+^]** 385.2106

Mass spectrum of **9a**

| **Formula** | **Calculated mass** | **Error / mDa** | **Error / ppm** | **DBE** |
| --- | --- | --- | --- | --- |
| C22 H29 N2 O4 | 385.2127 | -1.4327 | -3.7193 | 9.5 |
|  |  |  |  |  |

Expected: the same

**Figure S14.** NMR spectrum of **9b**

**Normorphine-β-*N*-ethylpiperidine (9b, C_23_H_30_N_2_O_3_)**

Yield: 62%

**^1^H NMR (600 MHz, CDCl_3_**) δ 6.61 (d, 8.2 Hz, 1H), 6.43 (d, J = 8.1 Hz, 1H), 5.62 (ddt, J = 10.0, 3.1, 1.5 Hz, 1H), 5.21 (dt, J = 9.8, 2.7 Hz, 1H), 4.85 – 4.75 (m, 1H), 4.16 – 4.06 (m, 1H), 3.36 (dd, J = 6.2, 3.2 Hz, 1H), 2.84 (d, J = 18.5 Hz, 1H), 2.76 – 2.64 (m, 1H), 2.66 – 2.53 (m, 3H), 2.51 (t, J = 7.5 Hz, 2H), 2.35 – 2.22 (m, 1H), 2.16 (td, J = 12.3, 3.3 Hz, 1H), 1.96 (td, J = 12.6, 5.0 Hz, 1H), 1.73 (dt, J = 12.3, 2.6 Hz, 1H), 1.67 ­– 1.52 (m, 6H), 1.45 (d, J = 8.8 Hz, 3H), 1.24 (s, 1H), 0.93 (s, 1H)

**APT NMR (151 MHz, CDCl_3_)** δ 145.94, 138.28, 133.02, 130.88, 128.52, 126.31, 119.70, 117.58, 91.49, 67.28, 66.65, 58.33, 54.87, 52.75, 44.56, 43.55, 40.71, 35.61, 25.44, 24.11, 21.85

**mp:** 80-82˚C **MS**: calculated: 383.5038, measured: **HR-MS [M+ H^+^]** 383.7118

Mass spectrum of **9b**

| **Formula** | **Calculated mass** | **Error / mDa** | **Error / ppm** | **DBE** |
| --- | --- | --- | --- | --- |
| C23 H31 N2 O3 | 383.2334 | -0.9681 | -2.5263 | 9.5 |
| C23 H31 N2 O4 | 399.2283 | -0.3828 | -0.9588 | 9.5 |

Expected: the same

**Figure S15.** NMR spectrum of **9c**

**Normorphine-β-*N*-ethylpyrrolidine (9c, C_22_H_28_N_2_O_3_)**

Yield: 68%

**^1^H NMR (600 MHz, CDCl_3_**) δ 6.63 (d, J = 8.0 Hz, 2H), 6.43 (d, J = 8.1 Hz, 1H), 5.64 (d, J = 9.8 Hz, 2H), 5.20 (dt, J = 9.8, 2.6 Hz, 2H), 4.87 – 4.74 (m, 2H), 4.50 – 3.90 (s, 13H), 4.12 (dt, J = 5.1, 2.6 Hz, 2H), 3.37 (dd, J = 6.4, 3.2 Hz, 2H), 3.33 – 3.23 (m, 1H), 2.87 – 2.71 (m, 7H), 2.70 – 2.62 (m, 2H), 2.62 – 2.55 (m, 3H), 2.32 – 2.19 (m, 3H), 2.05 – 1.94 (m, 4H), 1.96 – 1.82 (m, 7H), 1.25 (s, 2H), 1.21 – 1.12 (m, 2H), 1.12 (dd, J = 8.3, 6.4 Hz, 4H)

**APT NMR (151 MHz, CDCl_3_)** δ 145.96, 138.81, 133.24, 130.81, 127.89, 125.89, 119.72, 117.51, 90.82, 67.45, 66.56, 58.15, 54.14, 53.36, 45.49, 43.42, 40.69, 35.56, 23.29, 21.89

**mp:** 68-71˚C, **MS**: calculated: 369.4772, measured: **HR-MS [M+ H^+^]** 369.2150

Mass spectrum of **9c**

| **Formula** | **Calculated mass** | **Error / mDa** | **Error / ppm** | **DBE** |
| --- | --- | --- | --- | --- |
| C22 H29 N2 O3 | 369.2178 | -1.418 | -3.8408 | 9.5 |
| C22 H29 N2 O4 | 385.2127 | -1.1327 | -2.9405 | 9.5 |

Expected: the same

**Figure S16.** NMR spectrum of **10a**

**Normorphine-*N*-acetylmorpholine (10a, C_22_H_26_N_2_O_5_)**

Yield: 58%

**^1^H NMR (600 MHz, CDCl_3_)** δ 6.62 (d, J = 8.0 Hz, 1H), 6.45 (t, J = 8.2 Hz, 1H), 5.64 (d, J = 9.8 Hz, 1H), 5.21 (d, J = 9.9 Hz, 1H), 4.89 – 4.81 (m, 1H), 4.16 (d, J = 9.4 Hz, 2H), 3.74 – 3.62 (m, 9H), 3.61 (s, 2H), 3.48 – 3.30 (m, 2H), 3.27 (t, J = 4.9 Hz, 1H), 2.95 (d, J = 18.2 Hz, 1H), 2.58 (s, 1H), 2.52 – 2.45 (m, 1H), 2.35 (d, J = 6.9 Hz, 1H), 1.98 (td, J = 12.6, 5.3 Hz, 1H), 1.23 (s, 1H)

**APT NMR (151 MHz, CDCl_3_)** δ 168.69, 145.26, 138.10, 133.14, 130.57, 128.17, 126.07, 119.98, 116.96, 91.39, 66.49, 66.21, 57.01, 46.37, 44.81, 43.28, 40.88, 35.57, 21.69

**mp:**144-146˚C **MS**: calculated: 399.4601, measured: **HR-MS [M+ H^+^]** 399.1731.

Mass spectrum of **10a**

| **Formula** | **Calculated mass** | **Error / mDa** | **Error / ppm** | **DBE** |
| --- | --- | --- | --- | --- |
| C22 H27 N2 O5 | 399.1919 | -1.2972 | -3.2498 | 10.5 |
| C6 H16 N | 102.1282 | 0.0253 | 0.2482 | -0.5 |
| C31 H35 N4 O5 | 543.2607 | 0.4543 | 0.8363 | 16.5 |

Expected: the same

**Figure S17.** NMR spectrum of **10b**

**Normorphine-*N*-acetylpiperidine (10b, C_23_H_28_N_2_O_4_)**

Yield: 63%

**^1^H NMR (600 MHz, CDCl_3_)** δ 8.01 (s, 1H), 6.65 (d, J = 8.1 Hz, 1H), 6.47 (d, J = 8.1 Hz, 1H), 5.66 (ddt, J = 10.0, 3.1, 1.5 Hz, 1H), 5.24 (dt, J = 9.9, 2.7 Hz, 1H), 4.86 (dd, J = 6.5, 1.2 Hz, 1H), 4.20 – 4.15 (m, 1H), 3.62 – 3.46 (m, 5H), 3.44 – 3.36 (m, 1H), 3.38 (s, 1H), 3.32 (d, J = 13.2 Hz, 1H), 3.01 – 2.94 (m, 3H), 2.88 (s, 1H), 2.65 – 2.57 (m, 2H), 2.48 (td, J = 12.2, 3.5 Hz, 1H), 2.33 (dd, J = 18.7, 6.3 Hz, 1H), 2.01 (td, J = 12.4, 5.1 Hz, 1H), 1.84 (ddd, J = 12.6, 3.5, 1.8 Hz, 1H), 1.67 – 1.52 (m, 6H), 1.27 – 1.21 (m, 1H)

**APT NMR (151 MHz, CDCl_3_)** δ 168.38, 145.29, 138.05, 133.06, 128.36, 119.92, 116.89, 91.50, 66.15, 58.59, 56.98, 47.01, 44.78, 35.66, 26.77, 25.78, 24.47, 21.66.

**mp:**106-109˚C MS: calculated: 397.4873, measured: **HR-MS [M+ H^+^]** 397.2007

Mass spectrum of **10b**

| **Formula** | **Calculated mass** | **Error / mDa** | **Error / ppm** | **DBE** |
| --- | --- | --- | --- | --- |
| C23 H29 N2 O4 | 397.2127 | -2.5327 | -6.3763 | 10.5 |
|  |  |  |  |  |

Expected: the same

**Figure S18.** NMR spectrum of **10c**

**Normorphine-*N*-acetylpyrrolidine (10c, C_22_H_26_N_2_O_4_)**

Yield: 57%

**^1^H NMR (600 MHz, CDCl_3_)** δ 6.57 (d, 8.1 Hz, 1H), 6.39 (d, 8.2 Hz, 1H), 5.58 (m, J = 10.0, 3.0, 1.4 Hz, 1H), 5.12 (ddt, J = 12.4, 9.9, 2.7 Hz, 1H), 4.74 (ddd, J = 18.2, 6.5, 1.2 Hz, 1H), 4.04 (ddd, J = 12.5, 7.4, 4.2 Hz, 1H), 3.49 (t, J = 6.8 Hz, 2H), 3.48 – 3.32 (m, 5H), 3.18 (dt, J = 14.0, 6.3 Hz, 1H), 2.71 – 2.58 (m, 2H), 2.43 (m, J = 12.2, 3.4 Hz, 1H), 1.93 – 1.80 (m, 3H), 1.82 – 1.68 (m, 4H)

**APT NMR (151 MHz, CDCl_3_)** δ 168.88, 145.39, 138.14, 133.45, 130.86, 128.43, 126.19, 119.82, 116.93, 91.57, 66.57, 58.85, 57.26, 46.51, 43.30, 26.19, 24.09, 21.74

**mp:**108-110˚C **MS**: calculated: 383.4607, measured: **HR-MS [M+ H^+^]** 383.1874

Mass spectrum of **10c**

| **Formula** | **Calculated mass** | **Error / mDa** | **Error / ppm** | **DBE** |
| --- | --- | --- | --- | --- |
| C22 H27 N2 O4 | 383.197 | -2.2826 | -5.9569 | 10.5 |
| C22 H26 N2 O4 Na | 405.179 | -0.8273 | -2.0418 | 10.5 |

Expected: the same

**Figure S19.** NMR spectrum of **11a**

**Noroxymorphone-β-*N*-ethylmorpholine (11a, C_22_H_28_N_2_O_5_)**

Yield: 52%

**^1^H NMR (600 MHz, CDCl_3_)** δ 6.71 (d, J = 8.1 Hz, 1H), 6.58 (d, J = 8.2 Hz, 1H), 4.62 (s, 1H), 3.78 – 3.67 (m, 5H), 3.48 (s, 3H), 3.09 – 2.98 (m, 2H), 2.95 (d, J = 5.9 Hz, 1H), 2.70 – 2.60 (m, 4H), 2.57 – 2.47 (m, 2H), 2.37 (td, J = 12.4, 4.9 Hz, 1H), 2.31 – 2.20 (m, 2H), 1.85 (ddd, J = 13.4, 5.1, 2.9 Hz, 1H), 1.60 (td, J = 14.0, 3.4 Hz, 1H), 1.54 – 1.48 (m, 1H)

**APT NMR (151 MHz, CDCl_3_)** δ 210.05, 143.51, 139.06, 128.86, 123.86, 119.94, 118.29, 90.36, 76.77, 70.35, 66.81, 63.19, 56.69, 53.78, 51.00, 43.74, 36.13, 31.38, 30.58, 23.55

**mp:** 65-67˚C **MS**: calculated: 401.4760, measured: **HR-MS [M+ H^+^]** 401.0825

Mass spectrum of **11a**

| **Formula** | **Calculated mass** | **Error / mDa** | **Error / ppm** | **DBE** |
| --- | --- | --- | --- | --- |
| C22 H29 N2 O5 | 401.2076 | -1.8473 | -4.6045 | 9.5 |
| C22 H28 N2 O5 Na | 423.1895 | -1.492 | -3.5257 | 9.5 |
| C28 H40 N3 O6 | 514.2917 | -1.0114 | -1.9667 | 10.5 |
| C28 H39 N3 O6 Na | 536.2736 | -0.6561 | -1.2234 | 10.5 |

Expected: the same

**Figure S20.** NMR spectrum of **11b**

**Noroxymorphone-β-*N*-ethylpiperidine (11b, C_23_H_30_N_2_O_4_)**

Yield: 61%

**^1^H NMR (600 MHz, CDCl_3_)** δ 6.64 (d, J = 8.1 Hz, 1H), 6.47 (d, J = 8.1 Hz, 1H), 4.53 (d, J = 5.3 Hz, 1H), 4.09 (q, J = 6.5 Hz, 1H), 3.98 (dq, J = 15.0, 8.8, 7.6 Hz, 1H), 3.81 (q, J = 6.7 Hz, 1H), 3.70 (dq, J = 13.5, 6.7 Hz, 1H), 3.04 – 2.93 (m, 1H), 2.96 – 2.86 (m, 1H), 2.83 (s, 1H), 2.79 – 2.65 (m, 2H), 2.61 (q, J = 7.4, 5.2 Hz, 1H), 2.58 (s, 3H), 2.66 – 2.41 (m, 3H), 2.37 (tt, J = 12.5, 5.5 Hz, 1H), 2.20 – 2.10 (m, 1H), 2.06 (td, J = 12.2, 3.7 Hz, 1H), 1.78 (dt, J = 13.4, 3.9 Hz, 1H), 1.64 (q, J = 5.7 Hz, 3H), 1.53 (td, J = 14.0, 3.5 Hz, 1H), 1.48 – 1.39 (m, 3H), 1.36 (dd, J = 12.7, 3.3 Hz, 1H), 1.30 – 1.16 (m, 1H)

**APT NMR (151 MHz, CDCl_3_)** δ 209.43, 143.48, 138.82, 129.00, 124.14, 119.79, 117.85, 90.40, 76.60, 70.30, 63.23, 54.78, 51.61, 50.84, 43.70, 36.18, 31.32, 30.70, 25.81, 24.28, 23.53

**mp:** 68-70˚C **MS**: calculated: 399.5032, measured: **HR-MS [M+ H^+^]** 399.1032

Mass spectrum of **11b**

| **Formula** | **Calculated mass** | **Error / mDa** | **Error / ppm** | **DBE** |
| --- | --- | --- | --- | --- |
| C23 H31 N2 O4 | 399.2283 | -1.9828 | -4.9666 | 9.5 |
| C25 H35 N2 O5 | 443.2545 | -2.7976 | -6.3115 | 9.5 |

Expected: the same

**Figure S21.** NMR spectrum of **11c**

**Noroxymorphone-β-*N*-ethylpyrrolidine (11c, C_22_H_28_N_2_O_4_)**

Yield: 57%

**^1^H NMR (600 MHz, CDCl_3_)** δ 6.66 (d, J = 8.0 Hz, 1H), 6.53 (d, J = 8.1 Hz, 1H), 5.42 (s, 1H), 4.57 (s, 1H), 3.07 – 2.96 (m, 2H), 2.93 (d, J = 5.8 Hz, 1H), 2.78 (q, J = 9.1, 8.7 Hz, 1H), 2.73 – 2.47 (m, 6H), 2.33 (td, J = 12.6, 5.0 Hz, 1H), 2.24 (d, J = 14.8 Hz, 1H), 2.16 (td, J = 12.4, 3.5 Hz, 1H), 1.86 – 1.71 (m, 5H), 1.59 (td, J = 13.9, 3.2 Hz, 1H), 1.45 (dd, J = 12.9, 3.2 Hz, 1H)

**APT NMR (151 MHz, CDCl_3_)** δ 209.71, 144.12, 140.27, 129.11, 123.47, 119.82, 118.71, 89.88, 70.48, 63.80, 54.24, 53.26, 50.63, 43.47, 36.16, 31.21, 30.56, 23.43, 23.25

**mp:**87-90˚C **MS**: calculated: 385.4766.measured: **HR-MS [M+ H^+^]** 385.1963

Mass spectrum of **11c**

| **Formula** | **Calculated mass** | **Error / mDa** | **Error / ppm** | **DBE** |
| --- | --- | --- | --- | --- |
| C22 H29 N2 O4 | 385.2127 | -2.3327 | -6.0557 | 9.5 |
|  |  |  |  |  |

Expected: the same

**Figure S22.** NMR spectrum of **12a**

**Noroxymorphone-*N*-acetylmorpholine (12a, C_22_H_26_N_2_O_6_)**

Yield: 73%,

**^1^H NMR (600 MHz, CDCl_3_)** δ 8.00 (s, 1H), 6.70 (d, J = 8.2, 1H), 6.57 (d, J = 8.2 Hz, 1H), 5.22 (s, 1H), 4.64 (s, 1H), 4.17 – 4.10 (m, 1H), 3.82 – 3.71 (m, 1H), 3.72 – 3.56 (m, 15H), 3.51 (dt, J = 9.5, 4.8 Hz, 2H), 3.43 – 3.24 (m, 3H), 3.16 (s, 1H), 3.12 – 2.99 (m, 2H), 2.96 (d, J = 5.7 Hz, 1H), 2.95 – 2.90 (m, 4H), 2.86 – 2.84 (m, 2H), 2.64 (ddd, J = 18.6, 8.8, 6.0 Hz, 1H), 2.55 (dd, J = 11.3, 4.5 Hz, 1H), 2.52 – 2.42 (m, 3H), 2.40 (td, J = 12.0, 11.5, 3.2 Hz, 1H), 2.36 – 2.22 (m, 2H), 1.87 (ddd, J = 13.5, 5.0, 2.9 Hz, 1H), 1.63 – 1.46 (m, 3H), 1.24 (d, J = 19.4 Hz, 1H)

**APT NMR (151 MHz, CDCl_3_)** δ 209.73, 169.37, 143.80, 139.57, 129.12, 123.48, 119.71, 118.46, 90.06, 76.57, 70.42, 63.36, 54.61, 53.36, 50.69, 43.56, 36.18, 31.23, 30.61, 23.46

amorphous, **MS**: calculated: 415.4595, measured: **HR-MS [M+ H^+^]** 415.2875

Mass spectrum of **12a**

| **Formula** | **Calculated mass** | **Error / mDa** | **Error / ppm** | **DBE** |
| --- | --- | --- | --- | --- |
| C22 H27 N2 O6 | 415.1869 | -1.6119 | -3.8824 | 10.5 |
| C22 H26 N2 O6 Na | 437.1688 | -1.4565 | -3.3318 | 10.5 |

Expected: the same

**Figure S23.** NMR spectrum of **12b**

**Noroxymorphone-*N*-acethylpiperidine (12b, C_23_H_28_N_2_O_5_)**

Yield: 68%

**^1^H NMR (600 MHz, CDCl_3_)** δ 6.68 (d, J = 8.1 Hz, 1H), 6.56 (d, J = 8.0 Hz, 1H), 4.61 (s, 1H), 3.08 – 2.95 (m, 2H), 2.94 (d, J = 5.8 Hz, 1H), 2.71 – 2.63 (m, 1H), 2.65 – 2.55 (m, 2H), 2.58 – 2.42 (m, 2H), 2.36 (td, J = 12.5, 5.2 Hz, 1H), 2.27 (dt, J = 14.5, 3.2 Hz, 1H), 2.20 (td, J = 12.1, 3.7 Hz, 1H), 1.83 (ddd, J = 13.4, 5.1, 3.0 Hz, 1H), 1.64 – 1.54 (m, 5H), 1.51 – 1.41 (m, 3H)

**APT NMR (151 MHz, CDCl_3_)** δ 221.31, 167.70, 144.71, 138.18, 130.26, 123.67, 118.29, 117.00, 93.60, 77.22, 70.32, 57.39, 50.74, 47.80, 44.29, 36.50, 31.45, 29.04, 25.56, 24.39

23.36, amorphous, **MS**: calculated: 413.4867, measured: **HR-MS [M+ H^+^]** 413.3057

Mass spectrum of **12b**

| **Formula** | **Calculated mass** | **Error / mDa** | **Error / ppm** | **DBE** |
| --- | --- | --- | --- | --- |
| C23 H29 N2 O5 | 413.2076 | -1.6473 | -3.9868 | 10.5 |
| C23 H28 N2 O5 Na | 435.1895 | -1.492 | -3.4284 | 10.5 |
| C46 H56 N4 O10 Na | 847.3894 | -2.1143 | -2.4951 | 20.5 |

Expected: the same

**Figure S24.** NMR spectrum of **12c**

**Noroxymorphone-*N*-acetylpyrrolidine (12c, C_22_H_26_N_2_O_5_)**

Yield: 81%

**^1^H NMR (600 MHz, CDCl_3_)** δ 6.71 (d, J = 8.2 Hz, 1H), 6.58 (d, J = 8.2 Hz, 1H), 6.10 – 6.05 (m, 1H), 5.47 (s, 1H), 4.67 (s, 1H), 3.50 (t, J = 7.0 Hz, 2H), 3.41 (t, J = 6.8 Hz, 2H), 3.35 (d, J = 15.4 Hz, 1H), 3.24 (d, J = 15.4 Hz, 1H), 3.11 – 3.00 (m, 3H), 2.68 – 2.58 (m, 2H), 2.52 (td, J = 12.2, 5.0 Hz, 1H), 2.43 (td, J = 11.7, 3.5 Hz, 1H), 2.27 (dt, J = 14.5, 3.2 Hz, 1H), 2.03 – 1.92 (m, 3H), 1.91 – 1.83 (m, 3H), 1.58 (td, J = 13.9, 3.4 Hz, 1H), 1.56 – 1.50 (m, 1H)

**APT NMR (151 MHz, CDCl_3_)** δ 209.77, 164.96, 143.46, 138.85, 129.02, 123.92, 119.86, 117.81, 90.54, 70.42, 62.81, 56.65, 50.68, 45.88, 44.41, 36.15, 31.37, 30.54, 26.19, 24.09

**mp:** 92-95˚C MS: calculated: 399.4601, measured: **HR-MS [M+ H^+^]** 399.1674

Mass spectrum of **12c**

| **Formula** | **Calculated mass** | **Error / mDa** | **Error / ppm** | **DBE** |
| --- | --- | --- | --- | --- |
| C22 H27 N2 O5 | 399.1919 | -1.3972 | -3.5003 | 10.5 |
| C22 H26 N2 O5 Na | 421.1739 | -1.1419 | -2.7113 | 10.5 |

Expected: the same

**Other Compounds NMR spectrum results**

NMR spectrum of **4a**

**Normorphine (4a, C_16_H_17_NO_3_)**

**^1^H NMR (600 MHz, DMSO-d_6_)**  8.80 (s, 3-OH), 6.46 (d, *J* = 8.0 Hz, H-2), 6.35 (d, *J* = 8.1 Hz, H-1), 5.55 (dp, *J* = 9.9, 1.5 Hz, H-7), 5.20 (dt, *J* = 9.8, 2.8 Hz, H-8), 4.91 (s, 6-OH), 4.67 (dd, *J* = 6.1, 1.3 Hz, H-5), 4.07 (dd, *J* = 6.1, 3.1 Hz, H-6), 3.72 (m, H-9), 2.88 (dd, *J* = 13.2, 4.7 Hz, H-16), 2.73 (m, H-10), 2.72 (m, H-16), 2.55 (p, *J* = 2.8 Hz, H-14), 1.92 (td, *J* = 13.0, 5.0 Hz, H-15), 1.68 (m, H-15), 1.20 (s, H-10)

**^13^C NMR (151 MHz, DMSO-d_6_)**  146.7 (C4), 139.2 (C3), 134.5 (C7), 130.7 (C12), 127.6 (C8), 124.7 (C11), 119.1 (C1), 117.1 (C2), 92.1 (C5), 66.4 (C6), 51.4 (C9), 43.6 (C13), 39.6 (C14), 37.9 (C16), 35.0 (C15), 29.2 (C10)

NMR spectrum of **4b**

**Norcodeine (4b, C_17_H_19_NO_3_)**

**^1^H NMR (600 MHz, DMSO-d_6_)**  6.60 (d, *J* = 8.2 Hz, H-2), 6.44 (d, *J* = 8.2 Hz, H-1), 5.52 (dp, *J* = 9.7, 1.5 Hz, H-7), 5.21 (dt, *J* = 9.8, 2.8 Hz, H-8), 4.64 (dd, *J* = 5.9, 1.4 Hz, H-5), 4.09 (dh, *J* = 5.4, 2.6 Hz, H-6), 3.72 (s, OCH_3_), 3.46 (dd, *J* = 5.9, 3.3 Hz, H-9), 2.71 (m, H-10), 2.67 (m, H-16), 2.65 (m, H-10), 2.46 (q, *J* = 2.8 Hz, H-14), 1.82 (td, *J* = 12.2, 5.5 Hz, H-15), 1.59 (dt, *J* = 12.3, 2.4 Hz, H-15)

**^13^C NMR (151 MHz, DMSO-d_6_)**  147.7 (C4), 141.6 (C3), 133.7 (C7), 131.8 (C12), 129.1 (C8), 128.3 (C11), 118.7 (C1), 113.6 (C2), 93.1 (C5), 66.8 (C6), 56.5 (OCH_3_), 51.5 (C9), 44.3 (C13), 41.1 (C14), 38.4 (C16), 36.9 (C15), 31.3 (C10)

NMR spectrum of **4c**

**Dihydronorcodeine (4c, C_17_H_21_NO_3_)**

**^1^H NMR (600 MHz, DMSO-d_6_)**  6.69 (d, *J* = 8.1 Hz, H-2), 6.53 (d, *J* = 8.1 Hz, H-1), 4.43 (d, *J* = 4.9 Hz, H-5), 3.81 (m, H-6), 3.76 (s, OCH_3_), 3.17 (s, H-9), 2.81 (dd, *J* = 18.1, 6.2 Hz, H-10), 2.61 (m, H-10), 2.59 (m, H-16), 2.54 (m, H-16), 2.06 (ddd, *J* = 11.8, 6.7, 2.8 Hz, H-14), 1.66 (td, *J* = 12.3, 5.2 Hz, H-15), 1.45 (m, H-15), 1.36 (m, H-8), 1.32 (m, H-7), 1.18 (m, H-7), 0.83 (tt, *J* = 11.9, 6.1 Hz, H-8)

**^13^C NMR (151 MHz, DMSO-d_6_)**  147.5 (C4), 141.2 (C3), 130.9 (C12), 127.8 (C11), 118.5 (C1), 114.4 (C2), 91.1 (C5), 66.3 (C6), 56.7 (OCH_3_), 52.3 (C9), 43.0 (C13), 39.1 (C14), 38.4 (C16), 38.1 (C15) , 30.5 (C10), 26.4 (C7), 20.2 (C8)

NMR spectrum of **7a**

**Noroxymorphone (7a, C_16_H_17_NO_4_)**

**^1^H NMR (600 MHz, DMSO-d_6_)**  9.14 (s, 3-OH), 6.52 (d, *J* = 8.1 Hz, H-2), 6.49 (d, *J* = 8.1 Hz, H-1), 5.22 (s, 14-OH), 4.64 (s, H-5), 2.97 (m, H-9), 2.91 (m, H-10), 2.90 (m, H-7), 2.83 (m, H-10), 2.62 (dd, *J* = 13.1, 4.6 Hz, H-16), 2.37 (td, *J* = 12.8, 3.6 Hz, H-16), 2.29 (dd, *J* = 12.3, 4.8 Hz, H-15), 2.07 (dt, *J* = 14.2, 3.2 Hz, H-7), 1.74 (ddd, *J* = 13.6, 5.2, 2.9 Hz, H-8), 1.42 (td, *J* = 14.0, 3.5 Hz, H-8), 1.15 (dd, *J* = 12.1, 3.2 Hz, H-15)

**^13^C NMR (151 MHz, DMSO-d_6_)**  209.1 (C6), 143.9 (C4), 139.8 (C3), 130.0 (C12), 124.4 (C11), 119.4 (C1), 117.6 (C2), 89.9 (C5), 70.1 (C14), 57.3 (C9), 50.9 (C13), 37.8 (C16), 36.3 (C7), 31.83 (C10), 31.77 (C8), 30.0 (C15)

NMR spectrum of **7b**

**Noroxycodone (7b, C_17_H_19_NO_4_)**

**^1^H NMR (600 MHz, DMSO-d_6_)**  6.70 (d, *J* = 8.2 Hz, H-2), 6.62 (d, *J* = 8.2 Hz, H-1), 5.17 (s, 14-OH), 4.70 (s, H-5), 3.78 (s, OCH_3_), 2.96 (m, H-10), 2.94 (m, H-9), 2.91 (m, H-7), 2.88 (m, H-10), 2.58 (dd, *J* = 13.3, 4.7 Hz, H-16), 2.34 (td, *J* = 12.6, 3.2 Hz, H-16), 2.30 (td, *J* = 12.0, 4.5 Hz, H-15), 2.08 (dt, *J* = 14.1, 3.2 Hz, H-7), 1.74 (ddd, *J* = 13.6, 5.1, 2.9 Hz, H-8), 1.40 (td, *J* = 14.1, 3.4 Hz, H-8), 1.14 (dd, *J* = 12.1, 3.0 Hz, H-15)

**^13^C NMR (151 MHz, DMSO-d_6_)**  208.9 (C6), 144.9 (C4), 142.3 (C3), 130.3 (C12), 126.8 (C11), 119.5 (C1), 115.1 (C2), 90.5 (C5), 70.1 (C14), 57.3 (C9), 56.8 (OCH_3_), 51.0 (C13), 37.9 (C16), 36.3 (C7), 32.2 (C10), 32.0 (C8), 30.2 (C15)

NMR spectrum of **8a**

**Noroxymorphone ethylene ketal (8a, C_18_H_21_NO_5_)**

**^1^H NMR (600 MHz, DMSO-d_6_)** δ 8.82 (s, 3-OH), 6.53 (d, *J* = 8.0 Hz, H-2), 6.43 (d, *J* = 8.1 Hz, H-1), 4.78 (s, 14-OH), 4.28 (s, H-5), 4.04 (td, *J* = 6.9, 4.6 Hz) and 3.91 (q, *J* = 6.8 Hz) and 3.76 (q, *J* = 7.0 Hz) and 3.69 (td, *J* = 6.6, 4.7 Hz) (CH_2_ ketal), 2.85 (dd, *J* = 17.7, 5.7 Hz, H-10), 2.80 (m, H-9), 2.79 (m, H-10), 2.52 (m, H-16), 2.39 (td, *J* = 12.7, 3.9 Hz, H-16), 2.09 (m, H-15), 2.05 (m, H-7), 1.36 (m, H-8), 1.23 (m, H-7), 1.05 (dd, *J* = 12.2, 3.5 Hz, H-15)

**^13^C NMR (151 MHz, DMSO-d_6_)**  145.1 (C4), 139.2 (C3), 131.1 (C12), 124.1 (C11), 118.0 (C1), 117.2 (C2), 108.6 (C6), 93.3 (C5), 69.8 (C14), 66.4 and 64.6 (ketal CH_2_), 57.7 (C9), 48.3 (C13), 37.9 (C16), 32.2 (C10), 31.0 (C15), 29.4 (C7), 29.2 (C8)

NMR spectrum of **8b**

**Noroxycodone ethylene ketal (8b, C_19_H_23_NO_5_)**

**^1^H NMR (600 MHz, DMSO-d_6_)**  6.70 (d, *J* = 8.1 Hz, H-2), 6.54 (d, *J* = 8.2 Hz, H-1), 4.87 (s, 14-OH), 4.29 (s, H-5), 3.98 (td, *J* = 6.9, 4.6 Hz) and 3.77 (q, *J* = 6.8 Hz) and 3.74 (q, *J* = 7.0 Hz) and 3.65 (td, *J* = 6.6, 4.7 Hz) (CH_2_ ketal), 2.85 (m, H-10), 2.79 (m, H-9), 2, 2.49 (m, H-16), 2.32 (td, *J* = 12.7, 3.9 Hz, H-16), 2.07 (m, H-15), 2.01 (m, H-7), 1.34 (m, H-8), 1.29 (m, H-7), 1.03 (dd, *J* = 12.3, 3.6 Hz, H-15)

**^13^C NMR (151 MHz, DMSO-d_6_)**  146.2 (C4), 142.2 (C3), 131.3 (C12), 126.3 (C11), 118.2 (C1), 114.9 (C2), 108.9 (C6), 93.6 (C5), 69.9 (C14), 66.3 and 64.7 (ketal CH_2_), 57.5 (C9), 56.9 (OCH_3_), 48.3 (C13), 37.9 (C16), 32.0 (C10), 30.9 (C15), 29.3 (C7), 29.1 (C8)

NMR spectrum of **9d**

**Norcodeine-β-*N*-ethylmorpholine (9d, C_23_H_30_N_2_O_4_)**

Yield: 48%

**^1^H NMR (600 MHz, CDCl_3_)** δ 6.80 (d, J = 8.0 Hz, 1H), 6.68 (d, J = 7.9 Hz, 1H), 5.80 (d, J = 10.0 Hz, 1H), 5.36 (d, J = 8.8 Hz, 1H), 4.96 (d, J = 5.9 Hz, 1H), 4.45 (s, 1H), 4.29 (s, 1H), 4.02 (s, 3H), 3.91 – 3.77 (m, 5H), 3.74 (s, 1H), 3.62 (s, 2H), 3.14 (s, 1H), 3.00 (s, 1H), 2.70 (s, 1H), 2.55 (t, J = 12.5 Hz, 1H), 2.12 (d, J = 14.2 Hz, 1H), 1.29 (s, 1H)

**APT NMR (151 MHz, CDCl_3_)** δ 147.53, 142.47, 135.62, 129.20, 124.63, 122.91, 119.67, 114.57, 99.54, 65.93, 63.62, 56.38, 55.55, 55.14, 52.18, 46.07, 44.04, 41.78, 37.59, 21.44

amorphous MS: calculated: 399.5032, measured: **HR-MS [M+ H^+^]** 399.2185

NMR spectrum of **9e**

**Norcodeine-β-*N*-ethylpiperidine (9e, C_24_H_32_N_2_O_3_)**

Yield: 57%

**^1^H NMR (600 MHz, CDCl_3_)** δ 6.62 (d, J = 8.1 Hz, 1H), 6.53 (d, J = 8.2 Hz, 1H), 5.68 (d, J = 9.9 Hz, 1H), 5.25 (d, J = 9.3 Hz, 1H), 4.86 (d, J = 6.6 Hz, 1H), 4.17 – 4.12 (m, 1H), 3.82 (d, J = 10.3 Hz, 1H), 3.81 (s, 2H), 3.69 (q, J = 7.0 Hz, 1H), 3.52 – 3.43 (m, 1H), 3.45 (s, 1H), 3.09 (s, 1H), 2.92 (d, J = 18.5 Hz, 2H), 2.86 (s, 2H), 2.82 (s, 3H), 2.69 (dd, J = 12.2, 4.6 Hz, 1H), 2.65 – 2.61 (m, 1H), 2.48 – 2.35 (m, 2H), 2.02 (td, J = 12.5, 4.8 Hz, 1H), 1.56 (s, 2H), 1.21 (dd, J = 8.1, 6.0 Hz, 1H)

**APT NMR (151 MHz, CDCl_3_)** δ 146.43, 142.24, 133.52, 131.33, 127.98, 126.99, 119.60, 113.43, 91.12, 66.58, 58.99, 57.33, 56.22, 46.39, 45.90, 45.19, 43.22, 40.62, 35.73, 26.18, 24.11, 21.70

**mp:** 205-208˚C, **MS**: calculated: 397.5304, measured: **HR-MS [M+ H^+^]** 397.2393

NMR spectrum of **9f**

**Norcodeine-β-*N*-ethylpyrrolidine (9f, C_23_H_30_N_2_O_3_)**

Yield: 46%

**^1^H NMR (600 MHz, CDCl_3_)** δ 6.64 (d, J = 8.1 Hz, 1H), 6.54 (d, J = 8.1 Hz, 1H), 5.73 – 5.65 (m, 1H), 5.26 (dt, J = 10.0, 2.7 Hz, 1H), 4.90 – 4.84 (m, 1H), 4.15 (dq, J = 6.6, 4.1, 2.9 Hz, 1H), 3.82 (s, 3H), 3.49 – 3.40 (m, 1H), 2.97 (d, J = 18.6 Hz, 1H), 2.85 – 2.76 (m, 1H), 2.74 – 2.54 (m, 9H), 2.51 – 2.29 (m, 2H), 2.03 (td, J = 12.5, 5.0 Hz, 1H), 1.81 (p, J = 7.6, 6.8 Hz, 2H), 1.25 (d, J = 18.4 Hz, 1H), 0.82 (s, 1H)

**APT NMR (151 MHz, CDCl_3_)** δ 145.96, 138.81, 133.24, 130.81, 127.89, 125.89, 119.72, 117.51, 90.82, 67.45, 66.56, 58.15, 54.14, 53.36, 45.49, 43.42, 40.69, 35.56, 23.29, 21.89

**mp:** 90-92 ˚C **MS**: calculated: 383.5038.measured: **HR-MS [M+ H^+^]** 383.2189

NMR spectrum of **9g**

**Dihydronorcodeine-β-*N*-ethylmorpholine (9g, C_23_H_32_N_2_O_4_)**

Yield: 71%

**^1^H NMR (600 MHz, CDCl_3_)** δ 6.64 (d, J = 8.2 Hz, 1H), 6.53 (d, J = 8.2 Hz, 1H), 4.51 (d, J = 5.3 Hz, 1H), 3.92 (ddd, J = 7.3, 5.3, 3.4 Hz, 1H), 3.79 (s, 3H), 3.63 (t, J = 4.7 Hz, 4H), 3.07 (dd, J = 5.9, 2.7 Hz, 1H), 2.86 (d, J = 18.4 Hz, 1H), 2.63 (ddd, J = 12.3, 8.8, 6.2 Hz, 1H), 2.57 – 2.41 (m, 4H), 2.43 – 2.30 (m, 1H), 2.22 – 2.08 (m, 2H), 1.78 (td, J = 12.4, 4.9 Hz, 1H), 1.59 (ddd, J = 12.4, 3.6, 1.7 Hz, 1H), 1.48 – 1.37 (m, 1H), 1.35 (dq, J = 18.3, 5.9 Hz, 1H), 1.06 – 0.96 (m, 1H)

**APT NMR (151 MHz, CDCl_3_)** δ 146.40, 141.39, 130.06, 126.67, 118.80, 113.11, 90.45, 67.41, 66.70, 58.67, 57.26, 56.15, 53.89, 52.27, 45.67, 42.49, 39.97, 37.28, 26.80, 21.10, 19.19

amorphous, **MS**: calculated: 401.5191, measured: **HR-MS [M+ H^+^]** 401.2375

NMR spectrum of **9h**

**Dihydronorcodeine-β-*N*-ethylpiperidine (9h, C_24_H_34_N_2_O_3_)**

Yield: 68%

**^1^H NMR (600 MHz, CDCl_3_)** δ 6.72 (d, J = 8.2, 1H), 6.62 (d, J = 8.2 Hz, 1H), 4.58 (d, J = 5.4 Hz, 1H), 4.02 (dq, J = 6.8, 3.3 Hz, 1H), 3.86 (d, J = 2.0 Hz, 3H), 3.31 (d, J = 5.4 Hz, 1H), 3.15 (s, 1H), 2.89 (d, J = 18.6 Hz, 1H), 3.04 – 2.75 (s, 11H), 2.68 (dd, J = 12.0, 4.6 Hz, 1H), 2.53 (dd, J = 18.5, 5.7 Hz, 1H), 2.37 – 2.23 (m, 2H), 1.91 (s, 3H), 1.92 – 1.87 (m, 2H), 1.72 – 1.66 (m, 1H), 1.60 – 1.52 (m, 3H), 1.50 – 1.41 (m, 2H), 1.15 – 1.05 (m, 1H)

**APT NMR (151 MHz, CDCl_3_)** δ 151.17, 141.92, 132.42, 127.44, 119.30, 113.43, 90.01, 69.04, 67.38, 58.98, 56.54, 55.14, 54.24, 52.19, 45.06, 40.32, 33.03, 26.94, 21.86, 18.56.

**mp:** 227-230 ˚C**, MS**: calculated: 399.5463, measured: **HR-MS [M+ H^+^]** 399.2623

NMR spectrum of **9i**

**Dihydronorcodeine-β-*N*-ethylpyrrolidine (9i, C_23_H_32_N_2_O_3_)**

Yield: 68%

**^1^H NMR (600 MHz, CDCl_3_)** δ 6.68 (d, J = 8.2 Hz, 1H), 6.58 (d, J = 8.2 Hz, 1H), 4.55 (d, J = 5.2 Hz, 1H), 3.98 (s, 1H), 3.84 (d, J = 5.9 Hz, 3H), 3.13 (d, J = 2.8 Hz, 1H), 2.92 – 2.88 (m, 1H), 2.80 (d, J = 5.0 Hz, 1H), 2.76 (dd, J = 16.8, 8.7 Hz, 1H), 2.71 – 2.61 (m, 7H), 2.58 (dd, J = 12.0, 4.1 Hz, 1H), 2.40 (dd, J = 18.3, 5.8 Hz, 1H), 2.27 – 2.20 (m, 1H), 2.20 – 2.15 (m, 1H), 1.80 – 1.68 (m, 4H), 1.64 (d, J = 12.2 Hz, 1H), 1.49 (dt, J = 14.2, 6.9 Hz, 1H), 1.40 (ddd, J = 18.2, 10.1, 5.5 Hz, 2H), 1.12 (dd, J = 14.6, 7.4 Hz, 1H), 1.08 – 1.00 (m, 1H)

**APT NMR (151 MHz, CDCl_3_)** δ 146.13, 141.55, 130.18, 126.58, 119.15, 113.20, 90.32, 67.12, 58.94, 56.31, 54.43, 53.62, 52.67, 45.89, 40.03, 37.07, 26.95, 23.29, 19.09

amorphous, **MS**: calculated: 385.5197, measured: **HR-MS [M+ H^+^]** 385.3490

NMR spectrum of **10d**

**Norcodeine-*N*-acetylmorpholine (10d, C_23_H_28_N_2_O_5_)**

Yield: 61%

**^1^H NMR (600 MHz, CDCl_3_)** δ 6.65 (d, J = 8.0 Hz, 1H), 6.65 (d, J = 8.1 Hz, 1H), 5.70 (ddt, J = 10.1, 3.1, 1.5 Hz, 1H), 5.26 (dt, J = 9.9, 2.7 Hz, 1H), 4.87 (dd, J = 6.5, 1.2 Hz, 1H), 4.16 (ddd, J = 6.7, 5.2, 2.8 Hz, 1H), 3.83 (s, 1H), 3.82 (s, 2H), 3.80 – 3.63 (m, 7H), 3.62 (dd, J = 7.1, 4.1 Hz, 2H), 3.44 – 3.31 (m, 3H), 3.02 (d, J = 18.6 Hz, 1H), 2.94 (s, 1H), 2.87 (s, 1H), 2.67 – 2.58 (m, 2H), 2.51 (td, J = 12.2, 3.4 Hz, 1H), 2.38 (dd, J = 18.7, 6.3 Hz, 1H), 2.01 (td, J = 12.5, 5.2 Hz, 1H), 1.91 – 1.85 (m, 1H)

**APT NMR (151 MHz, CDCl_3_)** δ 170.33, 146.08, 143.40, 142.60, 131.52, 128.11, 119.48, 114.00, 102.82, 92.28, 66.14, 59.00, 58.67, 56.64, 50.03, 47.24, 44.73, 43.88, 34.41, 27.49, 26.50.

amorphous, **MS**: calculated: 413.4867, measured: **HR-MS [M+ H^+^]** 413.6128

NMR spectrum of **10e**

**Norcodeine-*N*-acetylpiperidine (10e, C_24_H_30_N_2_O_4_)**

Yield: 87%

**^1^H NMR (600 MHz, CDCl_3_)** δ 6.65 (d, J = 8.2 Hz, 1H), 6.56 (d, J = 8.2 Hz, 1H), 5.73 – 5.67 (m, 1H), 5.26 (dt, J = 10.1, 2.7 Hz, 1H), 4.87 (dd, J = 6.6, 1.3 Hz, 1H), 4.16 (s, 1H), 3.82 (s, 3H), 3.54 (dq, J = 15.9, 10.1, 7.8 Hz, 6H), 3.44 (d, J = 13.3 Hz, 1H), 3.38 (d, J = 13.0 Hz, 1H), 3.04 (d, J = 18.7 Hz, 1H), 2.94 (s, 2H), 2.86 (s, 1H), 2.71 (s, 2H), 2.54 (t, J = 12.2 Hz, 1H), 2.41 (dd, J = 18.6, 6.4 Hz, 1H), 2.09 (s, 1H), 1.88 (d, J = 12.7 Hz, 1H), 1.64 (q, J = 5.4, 4.9 Hz, 3H), 1.60 (s, 2H), 1.63 – 1.57 (m, 1H), 1.55 (s, 1H), 1.24 (s, 1H)

**APT NMR (151 MHz, CDCl_3_)** δ 168.72, 146.97, 142.71, 134.00, 131.25, 128.56, 127.59, 119.95, 113.43, 91.95, 77.40, 66.57, 64.27, 59.52, 57.31, 47.55, 45.28, 43.40, 41.08, 36.11, 27.48, 26.32, 25.20, 22.17

amorphous, **MS**: calculated: 411.5139, measured: **HR-MS [M+ H^+^]** 411.4823

NMR spectrum of **10f**

**Norcodeine-*N*-acetylpyrrolidine (10f, C_23_H_28_N_2_O_4_)**

Yield: 83%

**^1^H NMR (600 MHz, CDCl_3_)** δ 6.64 (d, J = 8.2 Hz, 1H), 6.55 (d, J = 8.2 Hz, 1H), 5.68 (ddt, J = 10.0, 3.1, 1.5 Hz, 1H), 5.25 (dt, J = 9.9, 2.7 Hz, 1H), 4.87 (dd, J = 6.5, 1.3 Hz, 1H), 4.15 (s, 1H), 3.82 (s, 3H), 3.61 – 3.41 (m, 6H), 3.35 (d, J = 13.9 Hz, 1H), 3.26 (d, J = 13.9 Hz, 1H), 3.03 – 2.89 (m, 3H), 2.86 (s, 1H), 2.74 – 2.66 (m, 2H), 2.51 (td, J = 12.2, 3.5 Hz, 1H), 2.38 (dd, J = 18.6, 6.3 Hz, 1H), 2.09 (td, J = 12.4, 5.1 Hz, 1H), 2.01 – 1.89 (m, 3H), 1.91 – 1.79 (m, 3H)

**APT NMR (151 MHz, CDCl_3_)** δ 170.39, 146.42, 142.23, 133.29, 130.76, 128.22, 126.57, 119.84, 113.43, 91.12, 66.27, 64.80, 57.50, 56.32, 46.39, 45.98, 42.87, 40.33, 35.44, 26.19, 24.04, 21.88

amorphous, **MS**: calculated: 397.4873, measured: **HR-MS [M+ H^+^]** 397.5341

NMR spectrum of **10g**

**Dihydronorcodeine-*N*-acetylmorpholine (10g, C_23_H_30_N_2_O_5_)**

Yield: 72%

**^1^H NMR (600 MHz, CDCl_3_)** δ 6.65 (d, J = 8.0 Hz, 1H), 6.53 (d, J = 8.3 Hz, 1H), 5.69 (d, J = 10.3 Hz, 1H), 5.65 – 5.59 (m, 1H), 5.40 – 5.32 (m, 1H), 5.24 (dd, J = 10.1, 3.0 Hz, 1H), 5.11 (dp, J = 6.5, 3.3 Hz, 1H), 5.01 (t, J = 5.3 Hz, 1H), 4.99 (s, 1H), 4.91 – 4.82 (m, 1H), 4.16 (dd, J = 6.3, 3.1 Hz, 1H), 4.16 – 4.05 (m, 1H), 3.85 – 3.79 (m, 4H), 3.76 – 3.65 (m, 8H), 3.63 – 3.55 (m, 3H), 3.39 (t, J = 2.9 Hz, 1H), 3.37 (s, 1H), 3.38 – 3.30 (m, 1H), 3.01 (dd, J = 18.9, 3.6 Hz, 1H), 2.98 – 2.91 (m, 2H), 2.85 (d, J = 3.9 Hz, 1H), 2.65 (q, J = 2.9 Hz, 0H), 2.59 (dt, J = 10.0, 4.9 Hz, 2H), 2.57 – 2.45 (m, 1H), 2.37 (ddd, J = 18.7, 6.4, 3.1 Hz, 1H), 2.07 – 1.91 (m, 1H), 1.36 (td, J = 7.3, 3.4 Hz, 1H), 1.00 (td, J = 7.2, 3.4 Hz, 1H)

**APT NMR (151 MHz, CDCl_3_)** δ 170.33, 146.97, 141.50, 132.09, 127.55, 119.49, 113.26, 97.10, 69.69, 66.14, 58.67, 58.03, 56.64, 45.22, 44.73, 44.65, 43.88, 34.95, 30.19, 26.50, 25.30

amorphous, **MS**: calculated: 415.5026, measured: **HR-MS [M+ H^+^]** 415.6718

NMR spectrum of **10h**

**Dihydronorcodeine-*N*-acetylpiperidine (10h, C_24_H_34_N_2_O_3_)**

Yield: 71%

**^1^H NMR (600 MHz, CDCl_3_)** δ 6.72 (d, J = 8.2 Hz, 1H), 6.62 (d, J = 8.2 Hz, 1H), 4.58 (d, J = 5.3 Hz, 1H), 4.02 (s, 1H), 3.86 (s, 3H), 3.31 (s, 1H), 3.15 (s, 1H), 2.89 (d, J = 18.5 Hz, 5H), 2.71 – 2.65 (m, 2H), 2.53 (dd, J = 18.6, 5.4 Hz, 1H), 2.33 (t, J = 12.2 Hz, 1H), 2.26 (d, J = 8.8 Hz, 1H), 2.08 (s, 1H), 1.98 – 1.83 (m, 5H), 1.69 (d, J = 12.4 Hz, 1H), 1.55 (dd, J = 16.7, 9.7 Hz, 3H), 1.51 – 1.41 (m, 2H), 1.24 (s, 1H), 1.13 – 1.05 (m, 1H)

**APT NMR (151 MHz, CDCl_3_)** δ 151.17, 141.92, 132.42, 127.44, 119.30, 113.43, 90.01, 67.05, 59.02, 56.33, 54.33, 48.08, 45.09, 39.99, 36.75, 29.49, 27.08, 21.86, 18.93

**mp:**227-230˚C **MS**: calculated: 399.5463, measured: **HR-MS [M+ H^+^]** 399.2623

NMR spectrum of **10i**

**Dihydronorcodeine-*N*-acetylpyrrolidine (10i, C_23_H_30_N_2_O_4_)**

Yield: 81%

**^1^H NMR (600 MHz, CDCl_3_)** δ 6.67 (d, J = 8.2 Hz, 1H), 6.58 (d, J = 8.2 Hz, 1H), 4.54 (d, J = 5.2 Hz, 1H), 4.00 – 3.94 (m, 1H), 3.82 (d, J = 1.1 Hz, 3H), 3.56 – 3.41 (m, 4H), 3.28 (d, J = 14.0 Hz, 1H), 3.20 – 3.11 (m, 2H), 2.90 (d, J = 18.4 Hz, 1H), 2.57 (dd, J = 12.1, 4.8 Hz, 1H), 2.42 (dd, J = 18.4, 5.8 Hz, 1H), 2.29 (td, J = 12.1, 3.6 Hz, 1H), 2.23 (ddd, J = 11.9, 5.8, 2.8 Hz, 1H), 1.96 – 1.77 (m, 5H), 1.65 – 1.59 (m, 1H), 1.51 – 1.35 (m, 3H), 1.10 – 1.00 (m, 1H)

**APT NMR (151 MHz, CDCl_3_)** δ 168.42, 146.15, 141.37, 130.21, 126.89, 118.49, 113.21, 90.01, 67.16, 58.12, 56.34, 42.39, 40.07, 37.21, 26.91, 24.05 21.35, 19.15

**mp:**129-130˚C **MS**: calculated: 399.5032, measured: **HR-MS [M+ H^+^]** 399.2144

NMR spectrum of **11d**

**Noroxycodone-β-*N*-ethylmorpholine (11d, C_23_H_30_N_2_O_5_)**

Yield: 75 %

**^1^H NMR (600 MHz, CDCl_3_)** δ 7.89 (s, 1H), 6.59 (d, J = 8.2 Hz, 1H), 6.53 (d, J = 8.2 Hz, 1H), 5.11 (s, 1H), 4.55 (s, 1H), 4.39 (d, J = 12.0 Hz, 1H), 3.79 (s, 1H), 3.78 (s, 3H), 3.68 – 3.52 (m, 5H), 2.99 (d, J = 18.4 Hz, 1H), 2.97 – 2.84 (m, 4H), 2.76 (s, 2H), 2.71 (s, 1H), 2.64 – 2.38 (m, 6H), 2.30 (td, J = 13.2, 12.7, 5.0 Hz, 1H), 2.20 – 2.10 (m, 2H), 1.75 (dt, J = 13.5, 3.9 Hz, 1H), 1.51 (td, J = 13.9, 3.2 Hz, 1H), 1.44 (dd, J = 12.7, 3.4 Hz, 1H)

**APT NMR (151 MHz, CDCl_3_)** δ 208.53, 144.83, 142.78, 129.40, 124.82, 119.36, 114.83, 90.18, 70.20, 66.81, 62.93, 56.66, 53.73, 50.91, 50.58, 43.73, 36.07, 31.40, 30.63, 23.45

**mp:** 98-102˚C MS: calculated: 415.5026.measured: **HR-MS [M+ H^+^]** 415.2223

NMR spectrum of **11e**

**Noroxycodone-β-*N*-ethylpiperidine (11e, C_24_H_32_N_2_O_4_)**

Yield: 68%

**^1^H NMR (600 MHz, CDCl_3_)** δ 8.00 (s, 1H), 6.68 (d, J = 8.1 Hz, 1H), 6.61 (d, J = 8.2 Hz, 1H), 4.64 (s, 1H), 3.88 (s, 3H), 3.11 – 2.93 (m, 3H), 2.94 (s, 3H), 2.86 (s, 2H), 2.68 – 2.53 (m, 4H), 2.46 (t, J = 6.9 Hz, 2H), 2.43 – 2.36 (m, 4H), 2.31 – 2.18 (m, 2H), 1.84 (ddd, J = 13.4, 5.1, 3.0 Hz, 1H), 1.64 – 1.50 (m, 6H), 1.43 (t, J = 6.4 Hz, 2H)

**APT NMR (151 MHz, CDCl_3_)** 208.59, 144.96, 142.92, 129.51, 124.90, 119.36, 114.85, 90.32, 70.26, 63.13, 57.14, 54.83, 51.62, 50.70, 43.83, 36.17, 31.52, 30.71, 25.90, 24.28, 23.53

amorphous, **MS**: calculated: 413.5298, measured: **HR-MS [M+ H^+^]** 413.2430

NMR spectrum of **11f**

**Noroxycodone-β-*N*-ethylpyrrolidine (11f, C_23_H_30_N_2_O_4_)**

Yield: 78%

**^1^H NMR (600 MHz, CDCl_3_)** δ 6.63 (d, J = 8.1 Hz, 1H), 6.57 (d, J = 8.1 Hz, 1H), 5.21 (s, 1H), 4.60 (s, 1H), 3.83 (s, 3H), 3.07 – 2.94 (m, 2H), 2.92 (d, J = 5.8 Hz, 1H), 2.74 – 2.53 (m, 9H), 2.42 (td, J = 12.6, 5.2 Hz, 1H), 2.25 – 2.12 (m, 2H), 1.84 – 1.77 (m, 1H), 1.55 (td, J = 13.9, 3.4 Hz, 1H), 1.49 (dd, J = 12.8, 3.6 Hz, 1H)

**APT NMR (151 MHz, CDCl_3_)** 208.46, 144.91, 142.86, 129.48, 124.85, 119.35, 114.87, 90.28, 76.89, 70.23, 63.31, 57.05, 54.30, 53.38, 50.61, 43.80, 36.12, 31.44, 30.65, 23.43

amorphous, **MS**: calculated: 399.5032, measured: **HR-MS [M+ H^+^]** 399.4032

NMR spectrum of **12d**

**Noroxycodone-*N*-acetylmorpholine (12d, C_23_H_28_N_2_O_6_)**

Yield: 59%

**^1^H NMR (600 MHz, CDCl_3_)** δ 7.97 (s, 1H), 6.68 (d, J = 8.1, 1H), 6.61 (d, J = 8.1 Hz, 1H), 4.81 – 4.73 (m, 1H), 4.71 – 4.61 (m, 1H), 4.13 (d, J = 1.2 Hz, 1H), 3.90 – 3.84 (m, 1H), 3.86 (s, 3H), 3.72 (t, J = 4.7 Hz, 2H), 3.68 (d, J = 4.5 Hz, 2H), 3.66 (d, J = 5.7 Hz, 3H), 3.67 – 3.55 (m, 6H), 3.55 – 3.45 (m, 2H), 3.39 – 3.30 (m, 1H), 3.26 (s, 2H), 3.25 (d, J = 9.8 Hz, 1H), 3.17 (d, J = 18.7 Hz, 1H), 3.07 – 2.97 (m, 2H), 2.92 (s, 2H), 2.84 (s, 1H), 2.84 (s, 2H), 2.76 (s, 3H), 2.64 – 2.50 (m, 4H), 2.45 (s, 1H), 2.25 (ddt, J = 15.1, 7.9, 3.6 Hz, 2H), 1.93 (s, 1H), 1.86 (dt, J = 13.7, 4.2 Hz, 0H), 1.63 – 1.49 (m, 3H), 1.21 (d, J = 6.7 Hz, 3H)

**APT NMR (151 MHz, CDCl_3_)** δ 209.47, 170.33, 145.58, 143.60, 130.59, 124.88, 118.85, 115.08, 87.58, 70.10, 66.14, 62.31, 60.00, 56.64, 48.35, 43.88, 36.72, 31.84, 31.38, 23.27

**mp:**173-175˚C **MS**: calculated: 429.4861.measured: **HR-MS [M+ H^+^]** 429.5856

NMR spectrum of **12e**

**Noroxycodone-*N*-acetylpiperidine (12e, C_24_H_30_N_2_O_5_)**

Yield: 62%

**^1^H NMR (600 MHz, CDCl_3_)** δ 6.65 (d, J = 8.2 Hz, 1H), 6.58 (d, J = 8.2 Hz, 1H), 4.61 (s, 1H), 3.84 (s, 2H), 3.57 (dt, J = 12.0, 5.3 Hz, 1H), 3.51 – 3.30 (m, 4H), 3.28 (d, J = 14.3 Hz, 1H), 3.13 – 3.04 (m, 1H), 3.04 – 2.89 (m, 2H), 2.85 – 2.73 (m, 1H), 2.62 (dd, J = 18.6, 5.9 Hz, 1H), 2.52 (dd, J = 11.7, 4.8 Hz, 1H), 2.43 (td, J = 12.4, 5.0 Hz, 1H), 2.40 – 2.19 (m, 2H), 1.83 (ddd, J = 13.5, 5.0, 2.9 Hz, 1H), 1.68 – 1.54 (m, 3H), 1.51 (s, 1H), 1.12 (t, J = 7.3 Hz, 1H)

**APT NMR (151 MHz, CDCl_3_)** δ 208.39, 167.50, 144.92, 142.95, 129.32, 124.59, 119.44, 114.96, 90.25, 77.29, 70.42, 56.54, 50.38, 46.29, 44.22, 36.04, 31.40, 30.48, 25.51, 24.37, 23.67

amorphous, **MS**: calculated: 427.5133.measured: **HR-MS [M+ H^+^]** 427.4856

NMR spectrum of **12f**

**Noroxycodone-*N*-acetylpyrrolidine (12f, C_23_H_28_N_2_O_5_)**

Yield: 67%

**^1^H NMR (600 MHz, CDCl_3_)** δ 7.89 (s, 1H), 6.59 (d, J = 8.2 Hz, 1H), 6.54 (d, J = 8.2 Hz, 1H), 4.59 (s, 1H), 3.96 (s, 1H), 3.78 (s, 3H), 3.44 – 3.31 (m, 6H), 3.23 (d, J = 15.6 Hz, 1H), 3.18 (t, J = 6.8 Hz, 1H), 3.06 – 2.98 (m, 2H), 2.96 (td, J = 14.4, 5.1 Hz, 1H), 2.85 (s, 3H), 2.76 (s, 3H), 2.60 (td, J = 17.1, 15.5, 5.4 Hz, 2H), 2.46 (td, J = 12.5, 5.1 Hz, 1H), 2.31 (td, J = 12.0, 3.5 Hz, 1H), 2.15 (dt, J = 14.4, 3.3 Hz, 1H), 1.93 – 1.85 (m, 3H), 1.87 – 1.77 (m, 2H), 1.53 – 1.40 (m, 2H), 1.23 – 1.15 (m, 6H), 0.97 (s, 1H), 0.79 – 0.68 (m, 4H)

**APT NMR (151 MHz, CDCl_3_)** δ 208.58, 162.45, 144.84, 142.83, 129.37, 124.51, 119.44, 114.87, 90.20, 70.37, 63.07, 56.52, 50.25, 45.97, 44.45 .35.99, 31.37, 30.26, 26.04, 23.91

amorphous, **MS**: calculated: 413.4867, measured: **HR-MS [M+ H^+^]** 413.1856

**Supplemental information related to the docked ligands**

**Numbering scheme**

**Table S1: Used ligands**

| **Compound** | **Name** | **Activity** | **Ref** |
| --- | --- | --- | --- |
| 1 | N-ethylpiperidyl-normorphine | agonist | - |
| 2 | N-ethylmorpholyl-normorphine | agonist | - |
| 3 | N-ethylpiperidyl-noroxymorphone | antagonist | - |
| 4 | N-acethylmorpholyl-normorphine | agonist | - |
| 5 | N-acethylpyrrolidyl-noroxymorphone | antagonist | - |
| 6 | N-ethylmorpholyl-noroxymorphone | antagonist | - |
| 7 | N-acethylmorpholyl-noroxymorphone | antagonist | - |
| 8 | N-ethylpyrrolidyl-noroxymorphone | antagonist | - |
| 9 | N-acethylpiperidyl-noroxymorphone | antagonist | - |
| 10 | N-acethylpyrrolidyl-normorphine | agonist | - |
| 11 | N-acethylpiperidyl-normorphine | agonist | - |
| 12 | N-ethylpyrrolidyl-normorphine | agonist | - |
| 13 | AT076 | antagonist | ^1^ |
| 14 | Methylnaltexone (Relistor) | antagonist | ^2^ |
| 15 | Azidomorphine | agonist | ^3^, ^4^ |
| 16 | BNAP | antagonist | ^5^ |
| 17 | BNTX | antagonist | ^6^ |
| 18 | N-CPM-H2BAMO | antagonist | ^7^ |
| 19 | Butorphanol | partial agonist | ^8^ |
| 20 | Codeine epoxide | agonist | ^9^ |
| 21 | Codeine | agonist | ^10^ |
| 22 | Conorphone | partial agonist | ^11^ |
| 23 | Cyprodime | antagonist | ^12^ |
| 24 | 3-deoxy-clocinnamox | antagonist | ^10^ |
| 25 | 6-desoxymorphine | agonist | ^13^ |
| 26 | Ethoxymethopon | agonist | ^14^ |
| 27 | Ethylmorphine | agonist | ^15^ |
| 28 | 17-N-phenylethyl-14-O-phenylpropyl-noroxycodone | agonist | ^16^ |
| 29 | 17-N-allyl-14-O-phenylpropyl-noroxymorphone | agonist | ^16^ |
| 30 | 17-N-CPM-14-O-phenylpropyl-noroxymorphone | agonist | ^16^ |
| 31 | 17-N-CBM-14-O-phenylpropyl-noroxymorphone | agonist | ^16^ |
| 32 | 17-N-phenylethyl-14-O-phenylpropyl-noroxymorphone | agonist | ^16^ |
| 33 | 17-N-propyl-14-O-phenylpropyl-noroxymorphone | agonist | ^16^ |
| 34 | Dihydrocodeine | agonist | ^17^ |
| 35 | Dihydromorphine | agonist | ^18^ |
| 36 | N-methyl - 3-methoxy - 4,14-hydroxyl - 6-cianomorphinan | agonist | ^19^ |
| 37 | N-methyl - 3,4-methoxy - 14-hydroxyl - 6-cianomorphinan | agonist | ^19^ |
| 38 | N-methyl - 3,14-methoxy - 4-hydroxyl - 6-cianomorphinan | agonist | ^19^ |
| 39 | N-methyl - 3,4,14-methoxy - 6-acrylonitrile-morphinan | agonist | ^19^ |
| 40 | N-methyl - 3,4,14-methoxy - 6-cianomorphinan | agonist | ^19^ |
| 41 | N-methyl - 3,14-methoxy - 4,5-epoxy - 6-cianomorphinan | agonist | ^19^ |
| 42 | Hydrocodone | agonist | ^10^ |
| 43 | Hydromorphone | agonist | ^10^ |
| 44 | 14-O- 3-methoxyphenylacetyl-naltrexone | partial agonist | ^20^ |
| 45 | 14-O- 4-methoxyphenylacetyl-naltrexone | antagonist | ^20^ |
| 46 | 14-O- 4-chlorophenylacetyl-naltrexone | antagonist | ^20^ |
| 47 | levallorphan | antagonist | ^21^ |
| 48 | levorphanol | agonist | ^22^ |
| 49 | 14β-O-2'-pyridylcarboxy-naltrexone | antagonist | ^23^ |
| 50 | 14β-O-3'-pyridylcarboxy-naltrexone | antagonist | ^23^ |
| 51 | 14β-O-4'-pyridylcarboxy-naltrexone | antagonist | ^23^ |
| 52 | 14β-O-benzoylcarboxy-naltrexone | antagonist | ^23^ |
| 53 | 14β-O-3'-isoquinolylcarboxy-naltrexone | antagonist | ^23^ |
| 54 | 14β-O-2'-quinolylcarboxy-naltrexone | antagonist | ^23^ |
| 55 | 6β-O-3'-isoquinolylacetamido-naltrexone | partial agonist | ^24^ |
| 56 | 6α-O-2'-quinolylacetamido-naltrexone | partial agonist | ^24^ |
| 57 | 6β-O-2'-quinolylacetamido-naltrexone | partial agonist | ^24^ |
| 58 | 6α-O-3'-quinolylacetamido-naltrexone | partial agonist | ^24^ |
| 59 | 6β-O-3'-quinolylacetamido-naltrexone | partial agonist | ^24^ |
| 60 | 6α-O-3'-quinolylacetamido-naltrexone | partial agonist | ^24^ |
| 61 | 6β-O-3'-quinolylacetamido-naltrexone | partial agonist | ^24^ |
| 62 | 6α-O-2'-pyridylacetamido-naltrexone | partial agonist | ^24^ |
| 63 | 6β-O-2'-pyridylacetamido-naltrexone | partial agonist | ^24^ |
| 64 | 6α-O-3'-pyridylacetamido-naltrexone | partial agonist | ^24^ |
| 65 | 6β-O-3'-pyridylacetamido-naltrexone | partial agonist | ^24^ |
| 66 | 6α-O-4'-pyridylacetamido-naltrexone | partial agonist | ^24^ |
| 67 | 6β-O-4'-pyridylacetamido-naltrexone | partial agonist | ^24^ |
| 68 | 6α-O-benzamido-naltrexone | partial agonist | ^24^ |
| 69 | 6β-O-benzamido-naltrexone | partial agonist | ^24^ |
| 70 | 6α-O-3'-isoquinolylacetamido-naltrexone | partial agonist | ^24^ |
| 71 | 14β-O-3'-quinolylcarboxy-naltrexone | partial agonist | ^23^ |
| 72 | 14β-O-4' -naphtalylcarboxy-naltrexone | antagonist | ^23^ |
| 73 | (+)-trans-3-CPM-2,3,4,4aalpha,5,6,7,7aalpha-octahydro-1H-benzo[4,5]furo[3,2-e]isoquinolin -9-ol | agonist | ^25^ |
| 74 | 14-methoxy-5-methyl-morphinone | agonist | ^14^ |
| 75 | 14-methoxymetopon | agonist | ^14^ |
| 76 | methanocodeinone | agonist | ^26^ |
| 77 | methocinnamox | antagonist | ^10^ |
| 78 | 14-methoxymorphone | agonist | ^27^ |
| 79 | morphine | agonist | ^10^ |
| 80 | morphinone | agonist | ^28^ |
| 81 | nalbuphine | agonist | ^2^ |
| 82 | naldemedine | antagonist | ^29^ |
| 83 | nalmefene | antagonist | ^30^ |
| 84 | nalodeine | antagonist | ^31^ |
| 85 | naloxone | antagonist | ^10^ |
| 86 | naltrexone | antagonist | ^10^ |
| 87 | nicomorphine | agonist | ^32^ |
| 88 | norhydrocodone | agonist | ^33^ |
| 89 | normorphine | agonist | ^34^ |
| 90 | noroxymorphone | agonist | ^34^ |
| 91 | naloxonazine | antagonist | ^35^ |
| 92 | oxycodone | agonist | ^10^ |
| 93 | oxymorphamine | agonist | ^36^ |
| 94 | oxymorphazine | agonist | ^37^ |
| 95 | oxymorphazone | agonist | ^38^ |
| 96 | oxymorphone-4-nitrophenylhydrazone | agonist | ^39^ |
| 97 | oxymorphone | agonist | ^10^ |
| 98 | oxymorphone-phenylhydrazone | agonist | ^39^ |
| 99 | pentamorphone | agonist | ^40^ |
| 100 | pentazocine | partial agonist | ^41^ |
| 101 | N-propargyl-noroxymorphone | antagonist | ^42^ |
| 102 | N-propyl-noroxymorphone | antagonist | ^42^ |
| 103 | samidorphan | antagonist | ^43^ |
| 104 | TAMO (monomer) | agonist | ^44^ |
| 105 | thebaine | agonist | ^45^ |
| 106 | 14β-2'-pyridylacetamido-naltrexone | antagonist | ^46^ |

1.	Zaveri, N. T., Journigan, V. B. & Polgar, W. E. Discovery of the First Small-Molecule Opioid Pan Antagonist with Nanomolar Affinity at Mu, Delta, Kappa, and Nociceptin Opioid Receptors. *ACS Chem. Neurosci.* **6**, 646–657 (2015).

2.	Wentland, M. P. *et al.* Syntheses of novel high affinity ligands for opioid receptors. *Bioorganic Med. Chem. Lett.* **19**, 2289–2294 (2009).

3.	Horváth, K. & Wollemann, M. Azidomorphine is an agonist of high-affinity opioid receptor binding sites. *Neurochem. Res.* **11**, 1565–1569 (1986).

4.	Knoll, J. *et al.* GB1396663A_Original_document. (1975).

5.	Williams, D. A. *et al.* 6β-N-Heterocyclic Substituted Naltrexamine Derivative BNAP: A Peripherally Selective Mixed MOR/KOR Ligand. *ACS Chem. Neurosci.* **7**, 1120–1129 (2016).

6.	Portoghese, P. S., Sultana, M., Moe, S. T. & Takemori, A. E. Synthesis of Naltrexone-Derived δ-Opioid Antagonists. Role of Conformation of the δ Address Moiety. *J. Med. Chem.* **37**, 579–585 (1994).

7.	Bidlack, J. M., Frey, D. K., Kaplan, R. A., Seyed-Mozaffari, A. & Archer, S. Affinity labeling of μ opioid receptors by sulhydryl alkylating derivatives of morphine and morphinone. *Mol. Pharmacol.* **37**, 50–59 (1990).

8.	Fulton, B. S., Knapp, B. I., Bidlack, J. M. & Neumeyer, J. L. Synthesis and pharmacological evaluation of hydrophobic esters and ethers of butorphanol at opioid receptors. *Bioorganic Med. Chem. Lett.* **18**, 4474–4476 (2008).

9.	Konno, F. *et al.* PHARMACOLOGICAL PROPERTIES OF CODEINE-7,8-OXIDE (CODEINE EPOXIDE), A NEW METABOLITE OF CODEINE. *Japan. J. Pharmacol* 387–390 (1982).

10.	Lewis, J. W. & Husbands, S. M. 14-amino-4,5-epoxymorphinan derivatives and their pharmacological actions. *Top. Curr. Chem.* **299**, 93–119 (2011).

11.	Kotick, M. P., Leland, D. L., Polazzi, J. O. & Schut, R. N. Analgesic narcotic antagonists. 1. 8.beta.-Alkyl-, 8.beta.-acyl-, and 8.beta.-(tertiary alcohol)dihydrocodeinones and -dihydromorphinones. *J. Med. Chem.* **23**, 166–174 (1980).

12.	Schmidhammer, H., Burkard, W. P., Eggstein-aeppli, L. & Smith, C. F. Synthesis and Biological Evaluation of 14-alkoxymorphinans. 2. (-)-n-(cyclopropylmethyl)-4,14-dimethoxymorphinan-6-one, A Selective μ Opioid Receptor Antagonist. *J. Med. Chem.* **32**, 418–421 (1989).

13.	Cunningham, C. W. *et al.* Opioids and efflux transporters. Part 2: P-glycoprotein substrate activity of 3- and 6-substituted morphine analogs. *J. Med. Chem.* **51**, 2316–2320 (2008).

14.	Fürst, Z., Búzás, B., Friedmann, T., Schmidhammer, H. & Borsodi, A. Highly potent novel opioid receptor agonist in the 14-alkoxymetopon series. *Eur. J. Pharmacol.* **236**, 209–215 (1993).

15.	Enginar, H. *et al.* Synthesis, radiolabeling and biodistribution of a new opioid glucuronide derivative: Ethyl-morphine glucuronide (em-glu). *J. Radioanal. Nucl. Chem.* **292**, 667–673 (2012).

16.	Greiner, E. *et al.* Synthesis and biological evaluation of 14-alkoxymorphinans. 18.1 N-substituted 14-phenylpropyloxymorphinan-6-ones with unanticipated agonist properties: Extending the scope of common structure-activity relationships. *J. Med. Chem.* **46**, 1758–1763 (2003).

17.	Rowell, F. J., Seymour, R. A. & Rawlins, M. D. Pharmacokinetics of intravenous and oral dihydrocodeine and its acid metabolites. *Eur. J. Clin. Pharmacol.* **25**, 419–424 (1983).

18.	Gilbert, A. K., Hosztafi, S., Mahurter, L. & Pasternak, G. W. Pharmacological characterization of dihydromorphine, 6- acetyldihydromorphine and dihydroheroin analgesia and their differentiation from morphine. *Eur. J. Pharmacol.* **492**, 123–130 (2004).

19.	Ben Haddou, T. *et al.* Exploring pharmacological activities and signaling of morphinans substituted in position 6 as potent agonists interacting with the μ opioid receptor. *Mol. Pain* **10**, 1–11 (2014).

20.	Kumar, V. *et al.* Synthesis, biological evaluation, and SAR studies of 14β-phenylacetyl substituted 17-cyclopropylmethyl-7, 8-dihydronoroxymorphinones derivatives: Ligands with mixed NOP and opioid receptor profile. *Front. Psychiatry* **9**, 1–10 (2018).

21.	Kuz’mina, N. E., Osipova, E. S., Kuz’min, V. S. & Sitnikov, V. B. General model of the opiate pharmacophore 3. Regions of the opiate pharmacophore determining antagonistic properties of the opiate receptor ligands. *Russ. Chem. Bull.* **57**, 1285–1298 (2008).

22.	Hanessian, S., Parthasarathy, S., Mauduit, M. & Payza, K. The power of visual imagery in drug design. Isopavines as a new class of morphinomimetics and their human opioid receptor binding activity. *J. Med. Chem.* **46**, 34–48 (2003).

23.	Li, G., Aschenbach, L. C. K., He, H., Selley, D. E. & Zhang, Y. 14-O-Heterocyclic-substituted naltrexone derivatives as non-peptide mu opioid receptor selective antagonists: Design, synthesis, and biological studies. *Bioorganic Med. Chem. Lett.* **19**, 1825–1829 (2009).

24.	Li, G. *et al.* Design, synthesis, and biological evaluation of 6α- and 6β-N-heterocyclic substituted naltrexamine derivatives as μ opioid receptor selective Antagonists. *J. Med. Chem.* **52**, 1416–1427 (2009).

25.	Cheng, C. Y. *et al.* Synthesis and Opioid Activity of 7-Oxygenated 2,3,4,4a,5,6,7,7a-octahydro-1H-benzofuro[3,2-e]isoquinolin-9-ols. *J. Med. Chem.* **37**, 3121–3127 (1994).

26.	Kotick, M. P. Analgesic Narcotic Antagonists. 6. 1 7β,8β-Methano- and 7β,8β-Epoxydihydrocodeinone. *J. Med. Chem.* **24**, 722–726 (1981).

27.	Stavitskaya, L. & Coop, A. Most Recent Developments and Modifications of 14-alkylamino and 14-alkoxy-4,5-epoxymorphinan Derivatives. *Mini-Reviews Med. Chem.* **11**, 1002–1008 (2011).

28.	Todaka, T., Ishida, T., Kita, H., Narimatsu, S. & Yamano, S. Bioactivation of morphine in human liver: Isolation and identification of morphinone, a toxic metabolite. *Biol. Pharm. Bull.* **28**, 1275–1280 (2005).

29.	Inagaki, M. *et al.* Discovery of naldemedine: A potent and orally available opioid receptor antagonist for treatment of opioid-induced adverse effects. *Bioorganic Med. Chem. Lett.* **29**, 73–77 (2019).

30.	Dixon, R. *et al.* Nalmefene: Safety and Kinetics After Single and Multiple Oral Doses of a New Opioid Antagonist. *J. Clin. Pharmacol.* **27**, 233–239 (1987).

31.	Pasternak, G. W. & Pan, Y. X. Mu opioids and their receptors: Evolution of a concept. *Pharmacol. Rev.* **65**, 1257–1317 (2013).

32.	Lobbezoo, M. W., Van Rooy, H. H., Van Wijngaarden, I. & Soudijn, W. Opiate receptor binding of nicomorphine and its hydrolysis products in rat brain. *Eur. J. Pharmacol.* **82**, 207–211 (1982).

33.	Thompson, C. M. *et al.* Activation of G-Proteins by Morphine and Codeine Congeners: Insights to the Relevance of O- and N-Demethylated Metabolites at μ- and δ-Opioid Receptors. *J. Pharmacol. Exp. Ther.* **308**, 547–554 (2004).

34.	Köteles, I. & Hosztafi, S. Vaccines against Drug Abuse: Morphine-Hapten Design and Synthesis. *Proceedings* **41**, 48 (2019).

35.	Hahn, E. F. & Pasternak, G. W. Naloxonazine, a potent, long-lasting inhibitor of opiate binding sites. *Life Sci.* **31**, 1385–1388 (1982).

36.	Botros, S. *et al.* Opioid Agonist and Antagonist Activities of Peripherally Selective Derivatives of Naltrexamine and Oxymorphamine. *J. Med. Chem.* **32**, 2068–2071 (1989).

37.	Jacobson, A. E. Biological evaluation of compounds for their physical dependence potential and abuse liability. XIII. Drug Testing Program of the Committee on Problems of Drug Dependence, Inc. *NIDA Res. Monogr.* **95**, 556–577 (1989).

38.	Pasternak, G. W. & Hahn, E. F. Long-Acting Opiate Agonists and Antagonists: 14-Hydroxydihydromorphinone Hydrazones. *J. Med. Chem.* **23**, 674–676 (1980).

39.	Hahn, E. F., Itzhak, Y., Nishimura, S., Johnson, N. & Pasternak, G. W. Irreversible opiate agonists and antagonists. III. Phenylhydrazone derivatives of naloxone and oxymorphone. *J. Pharmacol. Exp. Ther.* **235**, (1985).

40.	Rudo, F. G. *et al.* Antinociceptive activity of pentamorphone, a 14-β-aminomorpinone derivative, compared to fentanyl and morphine. *Anesth. Analg.* **69**, 450–456 (1989).

41.	Albertson, N. F. & Wetterau, W. F. The synthesis of pentazocine. *Journal of Medicinal Chemistry* vol. 13 302–303 (1970).

42.	Toth, G., Kramer, M., Sirokman, F., Borsodi, A. & Ronai, A. Preparation OF [7, 8, 19, 20 - 3H] Naloxone of high specific activity. *J. Label. Compd. Radiopharm.* **19**, 1021–1030 (1982).

43.	Wentland, M. P. *et al.* Synthesis and opioid receptor binding properties of a highly potent 4-hydroxy analogue of naltrexone. *Bioorganic Med. Chem. Lett.* **15**, 2107–2110 (2005).

44.	Archer, S., Seyed-Mozaffari, A., Jiang, Q. & Bidlack, J. M. 14α,14′β-[Dithiobis[(2-oxo-2,1-ethanediyl)imino]]bis(7,8-dihydromorphinone) and 14α,14′β-[Dithiobis[(2-oxo-2,1-ethanediyl)imino]]bis-7,8-dihydro-N-(cyclopropyl-methyl)normorphinone: Chemistry and Opioid Binding Properties. *J. Med. Chem.* **37**, 1578–1585 (1994).

45.	Novak, B., Hudlicky, T., Reed, J., Mulzer, J. & Trauner, D. Morphine Synthesis and Biosynthesis-An Update. *Curr. Org. Chem.* **4**, 343–362 (2005).

46.	Zhang, Y., Elbegdorj, O., Yuan, Y., Beletskaya, I. O. & Selley, D. E. Opioid receptor selectivity profile change via isosterism for 14-O-substituted naltrexone derivatives. *Bioorganic Med. Chem. Lett.* **23**, 3719–3722 (2013).

**Table S2: Energy calculations for clustering**

| **compound** | **E_a** | **E_i** | **LE_a** | **LE_i** | **LEIAC_a** | **LEIAC_i** | **Ea-Ei** | **LEa-LEi** | **LEIACa-LEIACi** |
| --- | --- | --- | --- | --- | --- | --- | --- | --- | --- |
| 1 | -9.86892 | -9.74849 | -0.35246 | -0.34816 | -0.65793 | -0.69632 | -0.12043 | -0.00430 | 0.03839 |
| 2 | -9.34539 | -8.93127 | -0.33376 | -0.31897 | -0.62303 | -0.74427 | -0.41412 | -0.01479 | 0.12125 |
| 3 | -8.83552 | -7.73607 | -0.30467 | -0.26676 | -0.51974 | -0.59508 | -1.09945 | -0.03791 | 0.07535 |
| 4 | -7.04644 | -7.05469 | -0.24298 | -0.24327 | -0.44040 | -0.44092 | 0.00825 | 0.00028 | 0.00052 |
| 5 | -9.38273 | -8.85407 | -0.32354 | -0.30531 | -0.58642 | -0.55338 | -0.52866 | -0.01823 | -0.03304 |
| 6 | -7.00084 | -8.77645 | -0.24141 | -0.30264 | -0.38894 | -0.79786 | 1.77561 | 0.06123 | 0.40892 |
| 7 | -7.99842 | -7.96523 | -0.26661 | -0.26551 | -0.49990 | -0.79652 | -0.03319 | -0.00111 | 0.29662 |
| 8 | -8.09664 | -10.03812 | -0.28917 | -0.35850 | -0.42614 | -0.66921 | 1.94148 | 0.06934 | 0.24307 |
| 9 | -8.46516 | -9.55736 | -0.28217 | -0.31858 | -0.47029 | -0.86885 | 1.09220 | 0.03641 | 0.39856 |
| 10 | -8.45041 | -7.87086 | -0.30180 | -0.28110 | -0.60360 | -0.52472 | -0.57955 | -0.02070 | -0.07888 |
| 11 | -7.91826 | -7.56103 | -0.27304 | -0.26073 | -0.43990 | -0.42006 | -0.35723 | -0.01232 | -0.01985 |
| 12 | -10.10048 | -8.99341 | -0.37409 | -0.33309 | -0.67337 | -0.52902 | -1.10707 | -0.04100 | -0.14434 |
| 13 | -12.31982 | -12.19303 | -0.38499 | -0.38103 | -0.61599 | -0.67739 | -0.12679 | -0.00396 | 0.06140 |
| 14 | -8.16344 | -9.07895 | -0.31398 | -0.34919 | -0.58310 | -0.69838 | 0.91551 | 0.03521 | 0.11528 |
| 15 | -9.49652 | -8.55229 | -0.41289 | -0.37184 | -1.58275 | -0.57015 | -0.94423 | -0.04105 | -1.01260 |
| 16 | -11.36506 | -13.99479 | -0.28413 | -0.34987 | -0.37884 | -0.93299 | 2.62973 | 0.06574 | 0.55415 |
| 17 | -12.27507 | -12.22668 | -0.38360 | -0.38208 | -0.68195 | -1.11152 | -0.04839 | -0.00151 | 0.42957 |
| 18 | -11.69756 | -11.75185 | -0.40336 | -0.40524 | -0.83554 | -1.06835 | 0.05429 | 0.00187 | 0.23281 |
| 19 | -9.17913 | -10.66892 | -0.38246 | -0.44454 | -0.61194 | -1.18544 | 1.48979 | 0.06207 | 0.57349 |
| 20 | -8.88092 | -8.62490 | -0.38613 | -0.37500 | -0.98677 | -0.66345 | -0.25602 | -0.01113 | -0.32332 |
| 21 | -9.19459 | -8.25971 | -0.41794 | -0.37544 | -1.31351 | -0.82597 | -0.93488 | -0.04249 | -0.48754 |
| 22 | -9.50821 | -9.97395 | -0.35216 | -0.36941 | -0.59426 | -0.90672 | 0.46574 | 0.01725 | 0.31246 |
| 23 | -9.26808 | -10.72628 | -0.35646 | -0.41255 | -0.84255 | -1.53233 | 1.45820 | 0.05608 | 0.68977 |
| 24 | -14.17243 | -13.99272 | -0.40493 | -0.39979 | -0.70862 | -1.39927 | -0.17971 | -0.00513 | 0.69065 |
| 25 | -8.58224 | -7.69576 | -0.42911 | -0.38479 | -1.22603 | -0.76958 | -0.88648 | -0.04432 | -0.45646 |
| 26 | -7.98361 | -8.51834 | -0.31934 | -0.34073 | -0.61412 | -1.06479 | 0.53473 | 0.02139 | 0.45067 |
| 27 | -9.65024 | -9.33846 | -0.41958 | -0.40602 | -1.20628 | -0.71834 | -0.31178 | -0.01356 | -0.48794 |
| 28 | -14.97478 | -12.68564 | -0.38397 | -0.32527 | -0.71308 | -0.70476 | -2.28914 | -0.05870 | -0.00833 |
| 29 | -14.29354 | -12.49747 | -0.43314 | -0.37871 | -0.95290 | -0.83316 | -1.79607 | -0.05443 | -0.11974 |
| 30 | -14.53119 | -13.35245 | -0.42739 | -0.39272 | -0.90820 | -1.02711 | -1.17874 | -0.03467 | 0.11891 |
| 31 | -14.10935 | -13.22825 | -0.40312 | -0.37795 | -0.67187 | -1.10235 | -0.88110 | -0.02517 | 0.43048 |
| 32 | -15.50271 | -12.10800 | -0.40797 | -0.31863 | -0.81593 | -0.71224 | -3.39471 | -0.08933 | -0.10370 |
| 33 | -12.75238 | -11.83119 | -0.38644 | -0.35852 | -0.75014 | -1.18312 | -0.92119 | -0.02791 | 0.43298 |
| 34 | -9.21966 | -8.01648 | -0.41908 | -0.36439 | -1.84393 | -0.66804 | -1.20318 | -0.05469 | -1.17589 |
| 35 | -8.68879 | -7.59318 | -0.41375 | -0.36158 | -1.44813 | -0.75932 | -1.09561 | -0.05217 | -0.68881 |
| 36 | -9.53795 | -9.03034 | -0.39741 | -0.37626 | -0.86709 | -1.00337 | -0.50761 | -0.02115 | 0.13628 |
| 37 | -9.41666 | -9.30101 | -0.37667 | -0.37204 | -0.94167 | -0.62007 | -0.11565 | -0.00463 | -0.32160 |
| 38 | -10.43539 | -9.16502 | -0.41742 | -0.36660 | -1.15949 | -1.30929 | -1.27037 | -0.05081 | 0.14980 |
| 39 | -11.50662 | -9.66786 | -0.42617 | -0.35807 | -1.15066 | -0.87890 | -1.83876 | -0.06810 | -0.27177 |
| 40 | -10.68344 | -9.28980 | -0.41090 | -0.35730 | -1.06834 | -0.92898 | -1.39364 | -0.05360 | -0.13936 |
| 41 | -9.53884 | -8.12344 | -0.38155 | -0.32494 | -0.95388 | -0.81234 | -1.41540 | -0.05662 | -0.14154 |
| 42 | -9.21589 | -7.89806 | -0.41890 | -0.35900 | -1.53598 | -0.87756 | -1.31783 | -0.05990 | -0.65842 |
| 43 | -8.75631 | -8.03957 | -0.41697 | -0.38284 | -0.97292 | -0.89329 | -0.71674 | -0.03413 | -0.07964 |
| 44 | -14.85145 | -13.91819 | -0.41254 | -0.38662 | -0.78166 | -1.26529 | -0.93326 | -0.02592 | 0.48363 |
| 45 | -14.38349 | -13.99971 | -0.39954 | -0.38888 | -0.75703 | -1.16664 | -0.38378 | -0.01066 | 0.40962 |
| 46 | -14.31527 | -13.55267 | -0.40901 | -0.38722 | -0.75344 | -1.12939 | -0.76260 | -0.02179 | 0.37595 |
| 47 | -9.13660 | -9.14898 | -0.43508 | -0.43567 | -1.14208 | -0.83173 | 0.01238 | 0.00059 | -0.31035 |
| 48 | -8.45370 | -7.21356 | -0.44493 | -0.37966 | -1.20767 | -0.65578 | -1.24014 | -0.06527 | -0.55189 |
| 49 | -12.53371 | -13.17207 | -0.37981 | -0.39915 | -0.65967 | -1.09767 | 0.63836 | 0.01934 | 0.43800 |
| 50 | -11.24595 | -12.47055 | -0.34079 | -0.37790 | -0.80328 | -1.13369 | 1.22460 | 0.03711 | 0.33040 |
| 51 | -12.18608 | -12.91672 | -0.36928 | -0.39142 | -0.64137 | -1.84525 | 0.73064 | 0.02214 | 1.20387 |
| 52 | -12.38790 | -13.29886 | -0.37539 | -0.40300 | -0.65199 | -1.47765 | 0.91096 | 0.02760 | 0.82566 |
| 53 | -14.83218 | -14.28526 | -0.40087 | -0.38609 | -0.74161 | -1.42853 | -0.54692 | -0.01478 | 0.68692 |
| 54 | -13.15473 | -14.47403 | -0.35553 | -0.39119 | -0.59794 | -1.44740 | 1.31930 | 0.03566 | 0.84946 |
| 55 | -7.21220 | -13.07678 | -0.19492 | -0.35343 | -0.31357 | -0.76922 | 5.86458 | 0.15850 | 0.45565 |
| 56 | -8.78654 | -11.46046 | -0.23747 | -0.30974 | -0.33794 | -0.63669 | 2.67392 | 0.07227 | 0.29875 |
| 57 | -7.03452 | -12.95851 | -0.19012 | -0.35023 | -0.31975 | -0.80991 | 5.92399 | 0.16011 | 0.49016 |
| 58 | -9.26403 | -10.50722 | -0.25038 | -0.28398 | -0.38600 | -0.52536 | 1.24319 | 0.03360 | 0.13936 |
| 59 | -8.52683 | -12.95973 | -0.23045 | -0.35026 | -0.32796 | -0.71999 | 4.43290 | 0.11981 | 0.39203 |
| 60 | -9.55441 | -11.96531 | -0.25823 | -0.32339 | -0.41541 | -0.66474 | 2.41090 | 0.06516 | 0.24933 |
| 61 | -7.49662 | -13.36248 | -0.20261 | -0.36115 | -0.34076 | -0.70329 | 5.86586 | 0.15854 | 0.36253 |
| 62 | -9.78921 | -8.89936 | -0.29664 | -0.26968 | -0.42562 | -0.88994 | -0.88985 | -0.02697 | 0.46432 |
| 63 | -7.73246 | -12.40814 | -0.23432 | -0.37600 | -0.30930 | -0.72989 | 4.67568 | 0.14169 | 0.42059 |
| 64 | -10.40648 | -10.13050 | -0.31535 | -0.30698 | -0.54771 | -0.72361 | -0.27598 | -0.00836 | 0.17590 |
| 65 | -11.70305 | -12.23351 | -0.35464 | -0.37071 | -0.55729 | -1.01946 | 0.53046 | 0.01607 | 0.46217 |
| 66 | -7.69574 | -8.77650 | -0.23320 | -0.26595 | -0.34981 | -0.87765 | 1.08076 | 0.03275 | 0.52784 |
| 67 | -7.33991 | -11.84887 | -0.22242 | -0.35906 | -0.30583 | -0.78992 | 4.50896 | 0.13664 | 0.48410 |
| 68 | -9.66973 | -9.67179 | -0.29302 | -0.29308 | -0.50893 | -0.96718 | 0.00206 | 0.00006 | 0.45825 |
| 69 | -8.23636 | -12.56170 | -0.24959 | -0.38066 | -0.37438 | -0.78511 | 4.32534 | 0.13107 | 0.41073 |
| 70 | -9.84394 | -11.74344 | -0.26605 | -0.31739 | -0.39376 | -0.83882 | 1.89950 | 0.05134 | 0.44506 |
| 71 | -15.01427 | -14.32803 | -0.40579 | -0.38724 | -0.71497 | -1.19400 | -0.68624 | -0.01855 | 0.47904 |
| 72 | -15.12439 | -14.53440 | -0.40877 | -0.39282 | -0.72021 | -1.21120 | -0.58999 | -0.01595 | 0.49099 |
| 73 | -7.61875 | -9.34744 | -0.34631 | -0.42488 | -0.69261 | -0.84977 | 1.72869 | 0.07858 | 0.15715 |
| 74 | -9.20581 | -8.47261 | -0.38358 | -0.35303 | -0.92058 | -0.77024 | -0.73320 | -0.03055 | -0.15034 |
| 75 | -8.00844 | -8.02056 | -0.33369 | -0.33419 | -0.80084 | -0.80206 | 0.01212 | 0.00050 | 0.00121 |
| 76 | -9.57872 | -8.62075 | -0.41647 | -0.37482 | -0.95787 | -0.71840 | -0.95797 | -0.04165 | -0.23948 |
| 77 | -14.89142 | -14.45234 | -0.41365 | -0.40145 | -0.87597 | -1.31385 | -0.43908 | -0.01220 | 0.43788 |
| 78 | -9.03358 | -8.13411 | -0.39276 | -0.35366 | -1.00373 | -0.90379 | -0.89947 | -0.03911 | -0.09994 |
| 79 | -8.83810 | -7.81217 | -0.42086 | -0.37201 | -1.47302 | -0.78122 | -1.02593 | -0.04885 | -0.69180 |
| 80 | -8.79987 | -7.96140 | -0.41904 | -0.37911 | -1.25712 | -0.88460 | -0.83847 | -0.03993 | -0.37252 |
| 81 | -9.08962 | -10.41366 | -0.34960 | -0.40053 | -0.60597 | -0.94670 | 1.32404 | 0.05092 | 0.34072 |
| 82 | -10.74433 | -14.76008 | -0.25582 | -0.35143 | -0.39794 | -1.13539 | 4.01575 | 0.09561 | 0.73745 |
| 83 | -8.09836 | -10.93725 | -0.32393 | -0.43749 | -0.67486 | -0.84133 | 2.83889 | 0.11356 | 0.16646 |
| 84 | -10.12597 | -9.97137 | -0.42192 | -0.41547 | -1.44657 | -0.90649 | -0.15460 | -0.00644 | -0.54008 |
| 85 | -10.31198 | -10.06766 | -0.42967 | -0.41949 | -1.14578 | -1.00677 | -0.24432 | -0.01018 | -0.13901 |
| 86 | -8.25283 | -11.01096 | -0.33011 | -0.44044 | -0.58949 | -1.00100 | 2.75813 | 0.11033 | 0.41151 |
| 87 | -11.56650 | -11.19154 | -0.31261 | -0.30247 | -0.44487 | -0.69947 | -0.37496 | -0.01013 | 0.25461 |
| 88 | -8.44954 | -8.21228 | -0.40236 | -0.39106 | -1.20708 | -1.02654 | -0.23726 | -0.01130 | -0.18054 |
| 89 | -8.16476 | -7.64641 | -0.40824 | -0.38232 | -1.16639 | -0.84960 | -0.51835 | -0.02592 | -0.31679 |
| 90 | -8.83610 | -7.72713 | -0.42077 | -0.36796 | -1.26230 | -1.10388 | -1.10897 | -0.05281 | -0.15842 |
| 91 | -5.97174 | -12.93199 | -0.12441 | -0.26942 | -0.16588 | -0.47896 | 6.96025 | 0.14501 | 0.31308 |
| 92 | -9.73883 | -8.49784 | -0.42343 | -0.36947 | -1.08209 | -0.77253 | -1.24099 | -0.05396 | -0.30956 |
| 93 | -9.53274 | -7.92721 | -0.43331 | -0.36033 | -1.19159 | -0.66060 | -1.60553 | -0.07298 | -0.53099 |
| 94 | -4.45613 | -11.53632 | -0.10128 | -0.26219 | -0.12732 | -0.41201 | 7.08019 | 0.16091 | 0.28469 |
| 95 | -9.36000 | -8.25961 | -0.40696 | -0.35911 | -0.78000 | -0.58997 | -1.10039 | -0.04784 | -0.19003 |
| 96 | -11.81238 | -9.47586 | -0.36914 | -0.29612 | -0.62170 | -0.86144 | -2.33652 | -0.07302 | 0.23974 |
| 97 | -9.26354 | -8.38195 | -0.42107 | -0.38100 | -0.92635 | -0.93133 | -0.88159 | -0.04007 | 0.00497 |
| 98 | -10.08607 | -8.90952 | -0.34780 | -0.30722 | -0.67240 | -0.55685 | -1.17655 | -0.04057 | -0.11556 |
| 99 | -11.74587 | -10.11433 | -0.43503 | -0.37460 | -0.97882 | -0.59496 | -1.63154 | -0.06043 | -0.38386 |
| 100 | -9.09639 | -8.92687 | -0.43316 | -0.42509 | -0.90964 | -0.81153 | -0.16952 | -0.00807 | -0.09811 |
| 101 | -8.79174 | -9.13649 | -0.36632 | -0.38069 | -0.73265 | -1.01517 | 0.34475 | 0.01436 | 0.28252 |
| 102 | -10.18042 | -9.59382 | -0.42418 | -0.39974 | -1.01804 | -0.73799 | -0.58660 | -0.02444 | -0.28006 |
| 103 | -10.16681 | -11.52003 | -0.37655 | -0.42667 | -0.56482 | -0.76800 | 1.35322 | 0.05012 | 0.20318 |
| 104 | -10.76045 | -9.00101 | -0.41386 | -0.34619 | -0.97822 | -0.81827 | -1.75944 | -0.06767 | -0.15995 |
| 105 | -8.82005 | -8.82899 | -0.38348 | -0.38387 | -0.80182 | -0.80264 | 0.00894 | 0.00039 | 0.00081 |
| 106 | -12.25639 | -13.08702 | -0.37141 | -0.39658 | -0.64507 | -1.18973 | 0.83063 | 0.02517 | 0.54466 |
